# Proteomics and Transcriptomics Uncover Key Processes for Elasnin Tolerance in Methicillin-Resistant Staphylococcus aureus

**DOI:** 10.1128/msystems.01393-21

**Published:** 2022-01-25

**Authors:** Jordy Evan Sulaiman, Lexin Long, Pei-Yuan Qian, Henry Lam

**Affiliations:** a Department of Chemical and Biological Engineering, The Hong Kong University of Science & Technology, Clear Water Bay, Kowloon, Hong Kong, People’s Republic of China; b Department of Ocean Science and Hong Kong Branch of Southern Marine Science and Engineering Guangdong Laboratory, Guangzhou, The Hong Kong University of Science & Technology, Clear Water Bay, Kowloon, Hong Kong, People’s Republic of China; c Southern Marine Science and Engineering Guangdong Laboratory, Guangzhou, Guangdong, People’s Republic of China; UCSF

**Keywords:** MRSA, *S. aureus*, antibiotics, elasnin, tolerance, proteomics, transcriptomics

## Abstract

Elasnin is a new antibiofilm compound that was recently reported to have excellent activity against methicillin-resistant Staphylococcus aureus (MRSA) biofilms. In this study, we established that elasnin also has antibacterial activity against growing S. aureus planktonic cells. To explore elasnin’s potential as an antibiotic, we applied adaptive laboratory evolution (ALE) and produced evolved strains with elevated elasnin tolerance. Interestingly, they were more sensitive toward daptomycin and lysostaphin. Whole-genome sequencing revealed that all of the evolved strains possessed a single point mutation in a putative phosphate transport regulator. Subsequently, they exhibited increased intracellular phosphate (P_i_) and polyphosphate levels. Inhibition of the phosphate transport regulator gene changed the phenotype of the wild type to one resembling those observed in the evolved strains. Proteomics and transcriptomics analyses showed that elasnin treatment resulted in the downregulation of many proteins related to cell division and cell wall synthesis, which is important for the survival of growing exponential-phase cells. Other downregulated processes and factors were fatty acid metabolism, glycolysis, the two-component system, RNA degradation, and ribosomal proteins. Most importantly, transport proteins and proteins involved in oxidative phosphorylation and the phosphotransferase system were more upregulated in the evolved strain than in the ancestral strain, indicating that they are important for elasnin tolerance. Overall, this study showed that elasnin has antibacterial activity against growing S. aureus cells and revealed the altered processes due to elasnin treatment and those associated with its tolerance.

**IMPORTANCE** Besides the excellent antibiofilm properties of elasnin, we discovered that it can also kill growing methicillin-resistant Staphylococcus aureus (MRSA) planktonic cells. We subjected MRSA cells to an *in vitro* evolution experiment, and the resulting evolved strains exhibited increased elasnin tolerance, reduced growth rate, loss of pigmentation, and an increased proportion of small-colony formation, and they became more sensitive toward daptomycin and lysostaphin. Through multiomics analysis, we uncovered the affected processes in growing S. aureus planktonic cells following elasnin treatment, including the downregulation of cell wall synthesis, cell division, and some genes/proteins for the two-component system. These findings suggest that elasnin suppressed processes important for the cells’ survival and adaptation to environmental stresses, making it an ideal drug adjuvant candidate. Overall, our study provides new insights into the mechanism of elasnin in S. aureus planktonic cells and pointed out the potential application of elasnin in clinics.

## INTRODUCTION

Elasnin, a neutral, lipophilic compound extracted from Streptomyces noboritoensis, was first introduced as a human granulocyte elastase inhibitor that has low or no toxic side effects (1, 2). It was then suggested that elasnin and other structurally similar compounds (e.g., substituted-alpha pyrones) may be used as agents to treat chronic obstructive lung disease due to the destruction of lung tissue by leukocyte elastase (3, 4). More recently, it was reported that elasnin also exhibited antibiofilm properties and is suitable for controlling biofilm formation and biofouling in the marine environment (5, 6). For instance, elasnin could inhibit the biofilm formation of multiple strains of marine bacteria, such as Escherichia coli, Vibrio alginolyticus, Microbacterium esteraromaticum, and Staphylococcus aureus, and prevented the attachment of large biofouling organisms and larval settlement of Balanus amphitrite. The mechanism of elasnin activity against methicillin-resistant Staphylococcus aureus (MRSA) biofilms has been recently explored (7). Briefly, elasnin repressed the virulence regulon on MRSA biofilm cells and interfered with the cell division process by suppressing the production of newly synthesized extracellular polymeric substance (EPS), cell wall and membrane components, and peptidoglycan hydrolases required to complete cell wall formation. Collectively, these effects destroyed the biofilm matrix and released the cell wall-defective biofilm cells, which became more sensitive to β-lactam antibiotics. Several processes on MRSA biofilm cells were also found to be affected after elasnin treatment (compared to the untreated cells), such as the two-component system, phosphotransferase system (PTS), transmembrane transport (including ABC transporter expression), quorum sensing and biofilm formation, pathogenesis, and β-lactam resistance.

In this study, we found that in addition to elasnin’s biofilm inhibition and eradication activity against MRSA biofilms, it also has antibacterial activity and can kill growing S. aureus planktonic cells upon prolonged treatment. In addition, when plated onto agar, elasnin-treated S. aureus cells formed small-colony variants (SCVs), which are a subpopulation of slow-growing bacteria with distinct phenotypic and virulence traits (8). To elucidate the affected processes and potential elasnin targets on growing S. aureus cells, we employed the strategy of adaptive laboratory evolution (ALE), which has been frequently used to study the evolution of tolerance and resistance (9–15), and generated evolved strains with elevated elasnin tolerance. The evolved strains were characterized and subjected to whole-genome sequencing, proteomics, and transcriptomics analyses to uncover the mechanism for their increased elasnin tolerance.

## RESULTS

### Adaptive laboratory evolution generated elasnin-tolerant evolved S. aureus strains.

We observed that elasnin can kill growing exponential-phase MRSA with a monophasic killing curve, although it cannot effectively eliminate nongrowing stationary-phase cells upon prolonged treatment (see Fig. S1a in the supplemental material. When plated onto agar medium, a significant portion of the surviving cells after 21 h of elasnin treatment formed small-colony variants (SCVs), which are defined as colonies whose size is 5 times smaller (or whose radius is 2.23 times smaller) than the most common colony type (8, 16) (Fig. S1b and c). The formation of these small colonies was not observed on untreated cells, suggesting that these were nonstable SCVs (17) and that elasnin-treated cells have trouble replicating. This is consistent with the previously reported action mechanism of elasnin, which interfered with the cell division process of biofilm-associated MRSA cells (7).

10.1128/msystems.01393-21.1 FIG S1 Elasnin treatment of MRSA cells. (a) Time-kill curve of stationary-phase and exponential-phase MRSA cells with elasnin (50 μg/mL) (mean ± SEM; *n* = 3). (b) Small-colony variant (SCV) formation on MRSA cells after treatment with elasnin. WT MRSA cells were treated with elasnin (50 μg/mL) for 21 h. Cells were washed, serially diluted, and plated on MH agar (21-h incubation time). The image of the plate shown is a representative image from 3 biological replicates. (c) Enlarged picture of the middle region of the plate in panel b, showing the formation of both normal-size colonies and SCVs. Download FIG S1, JPG file, 0.8 MB. Copyright © 2022 Sulaiman et al. 2022 Sulaiman et al. https://creativecommons.org/licenses/by/4.0/ This content is distributed under the terms of the Creative Commons Attribution 4.0 International license.

**FIG 1:**
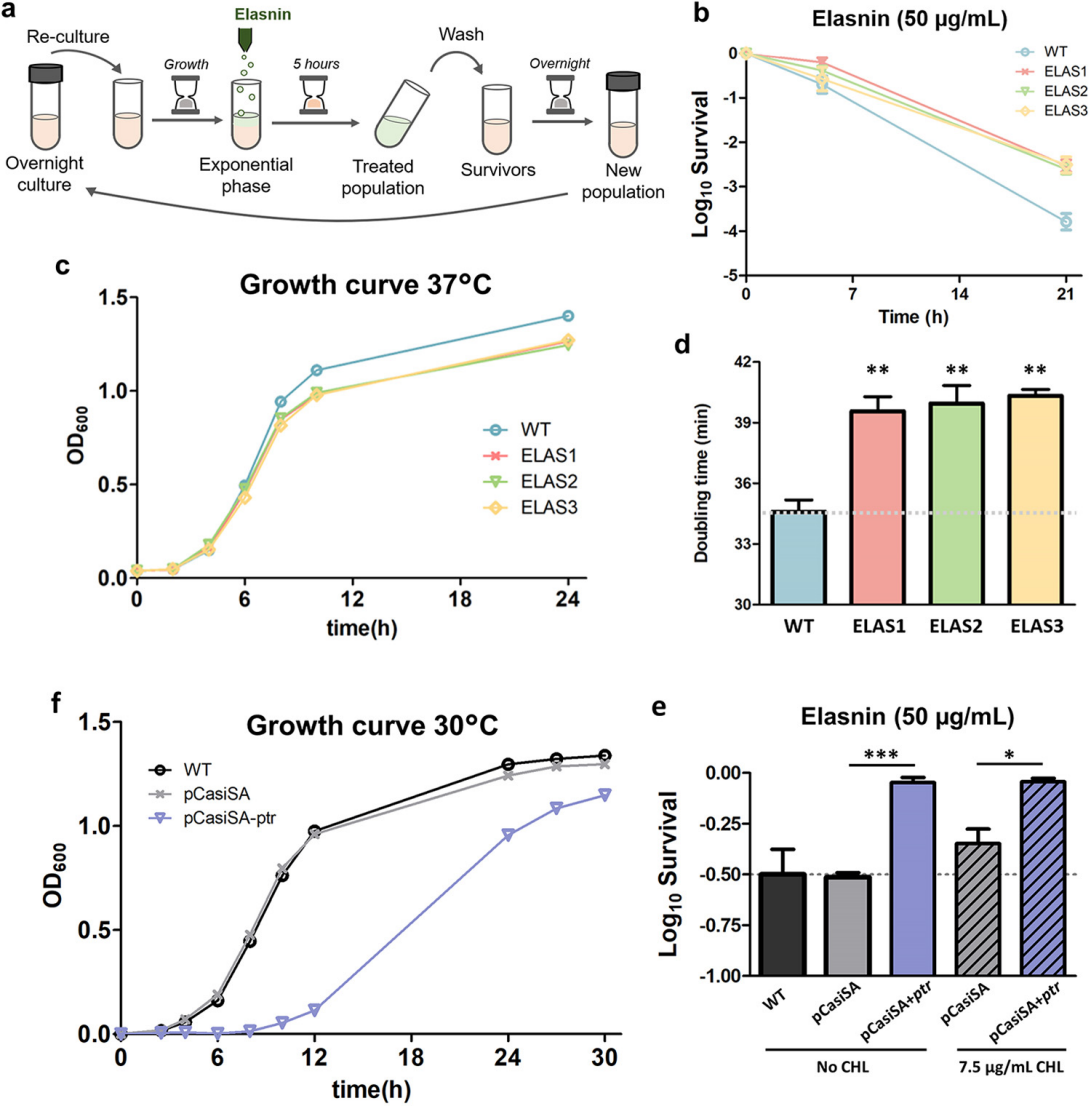
Laboratory evolution on MRSA using elasnin. (a) Experimental protocol for the adaptive evolution experiment on MRSA cells using elasnin. The cycle was repeated 7 times (1 week). (b) Time-kill curve of exponential-phase ancestral WT and evolved strains (ELAS1, ELAS2, and ELAS3) with elasnin (50 μg/mL) (mean ± SEM; *n* = 3). (c) Growth profile of ancestral WT and evolved strains (mean ± SEM; *n* = 3). (d) Doubling times of the ancestral WT and evolved strains, extracted from the fit to the exponential growth phase (mean ± SEM; *n* = 3). **, *P* < 0.01 (two-tailed Student’s *t* test for significance of difference from the ancestral strain). (e) Log_10_ survival of WT, pCasiSA, and pCasiSA-*ptr* strains in the absence and presence of 7.5 μg/mL chloramphenicol (CHL) upon treatment with elasnin (50 μg/mL) for 4 h (mean ± SEM; *n* = 3). The pCasiSA strain is the WT bearing empty pCasiSA plasmid, while the pCasiSA-*ptr* strain is the phosphate transport regulator-inhibited mutant. Significance of difference with the ancestral: ***, *P* < 0.001; *, *P* < 0.05 (two-tailed Student’s *t* test for significance of difference from the ancestral strain). f, Growth profile of WT, pCasiSA, and pCasiSA-*ptr* strains (mean ± SEM; *n* = 3).

To explore the affected processes of S. aureus planktonic cells upon treatment with elasnin, we first performed adaptive laboratory evolution (ALE) experiments on MRSA with elasnin to see how the cells adapt and evolve toward repetitive elasnin exposure. We treated the exponential-phase cells with a lethal dose of elasnin (50 μg/mL) daily for a week (Fig. 1a) and generated three evolved populations (ELAS1, ELAS2, and ELAS3) showing elevated tolerance toward elasnin, with around a 13- to 18-fold increase in survival after 21 h of treatment (Fig. 1b). The evolved populations had the same MIC toward elasnin as the ancestral strain (4 μg/mL), and therefore, the increased survival cannot be attributed to resistance. All of the evolved populations seemed to lose their characteristic orange pigment color (Fig. S2a). UV-visible (UV/Vis) spectrophotometry showed that the evolved strains had a shifted absorbance spectrum and a lower optical density at 450 nm (OD_450_; indicates the absorbance of staphyloxanthin pigment) than the ancestral strain (Fig. S2b and c). The dysregulation of pigment production in the elasnin-tolerant evolved strains hinted that they might have different fitness and virulence properties from the wild type (WT), since staphyloxanthin was known to act as a virulence factor (18, 19) and contributed to tolerance against oxidative stress by scavenging free radicals (20). Moreover, prolonging the growth of the cells up to 48 h still did not recover the pigment production in the evolved strains (Fig. S2d), suggesting that the loss-of-pigment phenotype is permanent rather than a result of temporary gene suppression. Growth measurement revealed that the evolved populations had slower growth than the ancestral strain (Fig. 1c), with a significant increase in the doubling time (Fig. 1d). This reinforces the notion that elasnin targets active growth processes, as evolved strains that grew slower could better survive elasnin treatment.

10.1128/msystems.01393-21.2 FIG S2 Elasnin-tolerant evolved strains and *ptr*-inhibited strain have reduced pigment production. (a) Visualization of the pigment color of WT ancestral, ELAS1, ELAS2, and ELAS3 strains after growth for 24 h. The circle above the tube shows the color of the cell pellet. (b) Absorbance spectrum of the carotenoids extracted from WT ancestral, ELAS1, ELAS2, and ELAS3 strains through methanol extraction (mean ± SEM; *n* = 3). (c) Measurement of the absorbance at 450 nm (OD_450_) of the carotenoids (mean ± SEM; *n* = 3). **, *P* < 0.01 (two-tailed Student’s *t* test for significance of difference from the ancestral WT). (d) Visualization of pigment on the WT and evolved strains after 24 h and 48 h of growth. For each of the WT and evolved strains in the 24-h and 48-h groups, 3 biological replicates are shown. The image shows that prolonging the growth for another 24 h does not recover the pigments on the evolved strains. (e) Visualization of the pigment color of the WT bearing empty pCasiSA plasmid and the pCasiSA-*ptr* strain. Three biological replicates are shown, and the circle above the tube shows the color of the cell pellet. On the right is shown the measurement of the OD_450_ of the carotenoids (mean ± SEM; *n* = 3). ***, *P* < 0.001, (two-tailed Student’s *t* test for significance of difference from the ancestral WT). Download FIG S2, TIF file, 2.0 MB. Copyright © 2022 Sulaiman et al. 2022 Sulaiman et al. https://creativecommons.org/licenses/by/4.0/ This content is distributed under the terms of the Creative Commons Attribution 4.0 International license.

### Whole-genome sequencing uncovered single point mutations in the elasnin-tolerant evolved strains.

To reveal the genetic basis of the elasnin-tolerant evolved populations, we isolated colonies from each of the evolved populations and sequenced their genomes (Table S1). Each of the evolved strains harbored two nonsynonymous single point mutations, with one common mutation that was shared across all strains, located in a hypothetical protein-coding sequence (E149K). Homology search of the hypothetical protein by BLAST showed a 100% identity match with a DUF47 domain-containing protein/phosphate transport regulator in other S. aureus strains, which belongs to the PhoU-like domain superfamily. Since this mutation was shared among the three evolved strains, and they all had similar tolerance levels, the same reduction in pigment production, and similar increases in doubling time, this mutation is likely the one that governs the observed tolerance phenotype in the evolved strains. The other mutations were point mutations in the *yjjP* gene (N215H) in ELAS1 and ELAS3, which codes for inner membrane protein YjjP, and in the *hisH* gene (R182L) in ELAS2, coding for imidazole glycerol phosphate synthase subunit HisH.

10.1128/msystems.01393-21.7 TABLE S1 List of nonsynonymous single point mutations detected by whole-genome sequencing. Download Table S1, DOCX file, 0.01 MB. Copyright © 2022 Sulaiman et al. 2022 Sulaiman et al. https://creativecommons.org/licenses/by/4.0/ This content is distributed under the terms of the Creative Commons Attribution 4.0 International license.

To verify that the putative phosphate transport regulator (*ptr*) gene is indeed the one that confers the observed phenotype on the evolved strains, we used the CRISPR/Cas9 transcription inhibition system pCasiSA to inhibit the expression of this gene (21) and compared the phenotype of the mutant with that of the WT. Interestingly, inhibition of *ptr* changed the phenotype of the WT to one resembling those observed in the evolved strains, namely, increased survival toward elasnin compared to that of the WT (Fig. 1e), slower growth in both the liquid culture (Fig. 1f) and agar plate (Fig. S3), and loss of pigmentation and reduced carotenoid production (Fig. S2e). Compared to the evolved strains, the survival of the *ptr*-inhibited mutant was somewhat higher after 4 h of elasnin treatment, with 90% survival (compared to 30% for the WT), whereas the survival of the evolved strains was in the range of 40% to 70% after 5 h of elasnin treatment (compared to 25% for the WT). This is consistent with the fact that the *ptr*-inhibited mutant had a much slower growth profile than the evolved strains (with doubling time of ∼110 min) (Fig. 1f). This indicated that elasnin tolerance is higher if the whole *ptr* gene is inhibited rather than a single point mutation as observed in the evolved strains. In addition, the *ptr*-inhibited mutant exhibited no increase in the MIC toward elasnin compared to the WT or the WT bearing empty pCasiSA (4 μg/mL). Together, these findings suggested that the mutation detected in the evolved strains reduced the expression of the *ptr* gene, which then resulted in the observed phenotype of increased tolerance.

10.1128/msystems.01393-21.3 FIG S3 Slow growth of colonies in pCasiSA-*ptr* strain. (a) Mid-exponential-phase WT and pCasiSA-*ptr* cultures were plated on MH agar. (Left) Representative plate of the WT and pCasiSA-*ptr* strain upon 24 h of growth; (right) representative plate of the WT and pCasiSA-*ptr* strain upon 48 h of growth. (b) Side-by-side plate comparison between the pCasiSA-*ptr* and WT strains shows a clear difference in size of the colonies after 48 h of growth in MH agar. Download FIG S3, JPG file, 0.9 MB. Copyright © 2022 Sulaiman et al. 2022 Sulaiman et al. https://creativecommons.org/licenses/by/4.0/ This content is distributed under the terms of the Creative Commons Attribution 4.0 International license.

### The evolved strains have increased levels of P_i_ and polyphosphate.

**FIG 2:**
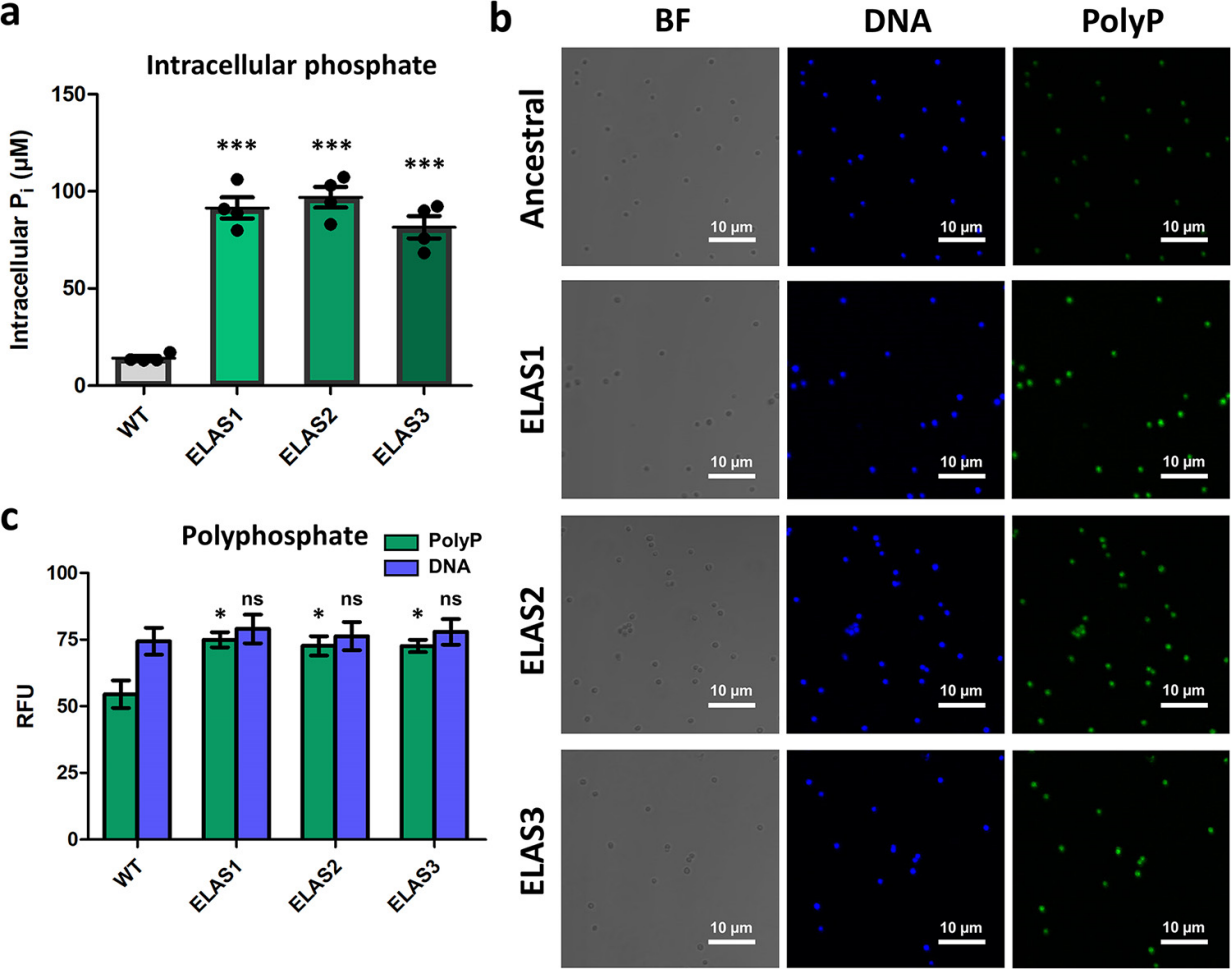
Increase in intracellular phosphate (P_i_) and polyphosphate levels in the elasnin-evolved strains. (a) Intracellular P_i_ concentrations were measured for the ancestral WT and elasnin-evolved strains (ELAS1, ELAS2, and ELAS3) (mean ± SEM; *n* = 4). ***, *P* < 0.001 (two-tailed Student’s *t* test for significance of difference from the ancestral strain). (b) Microscopic images of stationary-growth-phase wild-type ancestral and evolved strains. BF, bright field; DNA, stained with DAPI with emission at 461 nm; PolyP (polyphosphate), stained with DAPI with emission at 550 nm. (c) Quantification of fluorescence for polyP (at 415-nm excitation and 550-nm emission) and DNA (at 358-nm excitation and 461-nm emission) using a microplate reader (mean ± SEM; *n* = 3). *, *P* < 0.05 (two-tailed Student’s *t* test for significance of difference from the ancestral strain). ns, not significant.

Because all of the evolved strains have a mutation in the putative phosphate transport regulator, we were led to investigate whether intracellular phosphate (P_i_) levels were altered in the evolved strains. Interestingly, all of the elasnin-evolved strains (ELAS1, ELAS2, and ELAS3) had an elevated intracellular phosphate level, at least 6-fold, compared to the ancestral strain (Fig. 2a and Fig. S4). Because bacteria can store intracellular P_i_ as polyphosphate (polyP), forming chains made up of tens to hundreds of P_i_ moieties (22), we also measured the relative abundance of polyP in the cells. Confocal microscopy of 4′,6-diamidino-2-phenylindole (DAPI)-stained cells showed that the level of polyP appeared to be higher in the evolved strains than in the ancestral WT (Fig. 2b), which was further confirmed through microplate reader measurements (Fig. 2c).

10.1128/msystems.01393-21.4 FIG S4 Visualization of the reaction during intracellular phosphate measurement. Briefly, a commercial kit (ab65622; Abcam) was used to quantify intracellular phosphate (P_i_) levels. The same amounts of overnight ancestral WT and evolved strains were lysed using sonication, and the supernatant was mixed with the phosphate reagent. The measurement of phosphate relies on the reaction of malachite green and ammonium molybdate that forms a chromogenic complex with phosphate ion, showing an intense absorption band at OD measured at 650 nm. The darker green color of the mixture indicates a higher level of phosphate. The wells outlined with red mark the dilutions of the phosphate standard (0, 1, 2, 3, 4, and 5 nmol, from left to right) used to generate the standard curve. The wells outlined with blue, yellow, pink, and purple mark the first, second, third, and fourth biological replicates of the WT and evolved strains, respectively. For each replicate, different dilutions were prepared (marked on the leftmost column). it can be seen that after 30 min of incubation prior to adding the phosphate reagent to the samples, the evolved strains (ELAS1, ELAS2, and ELAS3) had a darker green color than the WT in all biological replicates. Download FIG S4, TIF file, 0.7 MB. Copyright © 2022 Sulaiman et al. 2022 Sulaiman et al. https://creativecommons.org/licenses/by/4.0/ This content is distributed under the terms of the Creative Commons Attribution 4.0 International license.

### Proteomic analysis of S. aureus upon elasnin treatment.

**FIG 3:**
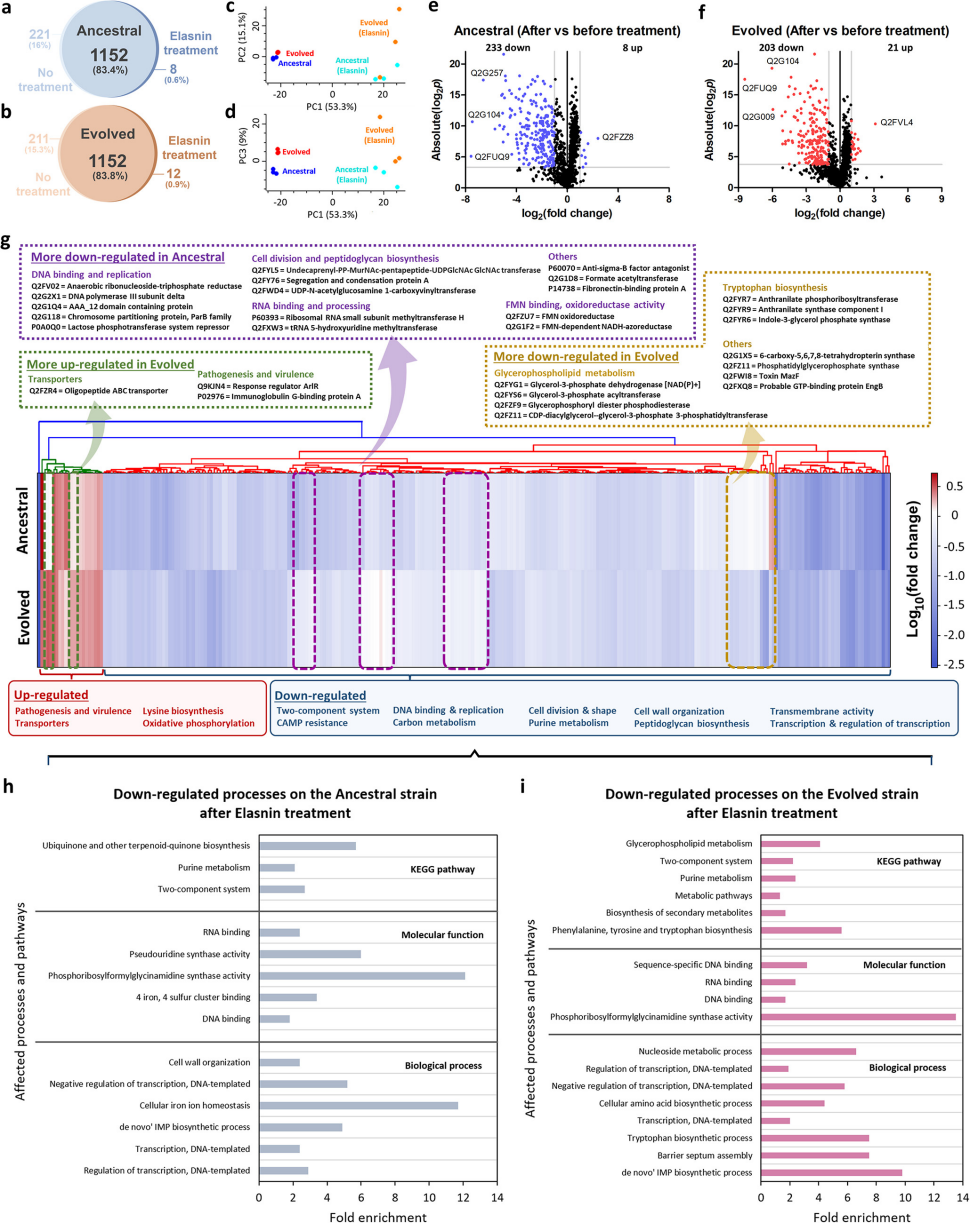
Proteomics analysis of the evolved and ancestral strains upon elasnin treatment. (a and b) Venn diagrams for proteome comparison of the ancestral WT (a) and the elasnin-evolved strain (b) before and after elasnin treatment (50 μg/mL) for 5 h. (c and d) Principal-component analysis (PCA) of proteomes: PC1 versus PC2 (c) and PC1 versus PC3 (d). (e and f) Volcano plots of the ancestral WT (e) and the elasnin-evolved strain (f) after elasnin treatment compared to those before treatment. Differentially expressed proteins (DEPs) were defined to be those with a permutation-based false-discovery rate (FDR) below 0.05, and absolute fold change greater than 2, corresponding to the colored dots. The protein identifiers (IDs) of the most downregulated and upregulated proteins are shown. (g) Heat map of log_10_ fold change of the DEPs across the ancestral and the evolved populations under elasnin treatment compared to the untreated populations. Hierarchical clustering was performed using Euclidean distance and a centroid linkage model. The upregulated and downregulated processes are listed below the heat map. The identities of some proteins with differential expression between the ancestral and evolved strains (outlined with green, purple, and yellow boxes) are listed. (h and i) Gene ontology (GO) analysis and pathway enrichment study (KEGG) by DAVID of the downregulated proteins of ancestral (h) and evolved (i) strains after elasnin treatment. Fold enrichment was defined as the ratio of the proportion of the input information to the background information.

We subjected the evolved strain (ELAS1) and the ancestral WT to quantitative proteomics, both in the absence and the presence of elasnin. Combining all replicates, 1,160 and 1,164 distinct proteins were identified for the elasnin-treated ancestral and evolved strain, respectively, whereas 1,373 and 1,363 distinct proteins were identified for the untreated ancestral and evolved strain, respectively (Fig. 3a and b). Using the protein expression data, we performed a principal-component analysis (PCA) to determine if there were protein features that distinguished the evolved and the ancestral strains both in the absence and presence of an antibiotic. Here, we focused on the first 3 PCs, as they captured more than 75% of the variation between samples. From the PCA plot, we could see that in the absence of the antibiotic, the proteomes of the evolved and ancestral strains were positioned similarly, while after elasnin treatment, the proteomes of the evolved and ancestral strains were separated from each other (Fig. 3c and d). Figure 3e and f show the volcano plots of fold changes against *P values* (two-tailed Student’s *t* test), along with the number of differentially expressed proteins (DEPs), highlighting proteins that were differentially expressed after elasnin treatment compared to those before treatment. The full list of DEPs is available in Table S2. From the volcano plots, it is evident that most of the proteins in both the ancestral and evolved strains were downregulated after 5 h of elasnin treatment.

10.1128/msystems.01393-21.8 TABLE S2 Differentially expressed proteins (DEPs) in the ancestral and evolved strain after elasnin treatment compared those before treatment. Download Table S2, XLSX file, 0.04 MB. Copyright © 2022 Sulaiman et al. 2022 Sulaiman et al. https://creativecommons.org/licenses/by/4.0/ This content is distributed under the terms of the Creative Commons Attribution 4.0 International license.

From the heat map of fold changes of the DEPs across the ancestral and evolved strains, we observed that most of the DEPs in the ancestral and evolved strains shared similar differential expression patterns upon elasnin treatment (Fig. 3g). The few proteins that were upregulated are transport proteins and those that play a role in pathogenesis, lysine biosynthesis, and oxidative phosphorylation, whereas proteins involved in the two-component system, cationic antimicrobial peptide (CAMP) resistance, cell wall organization and peptidoglycan biosynthesis, cell division, and transmembrane processes were mostly downregulated. As shown from the heat map, a few proteins were more upregulated in the evolved strain than in the ancestral strain. For instance, immunoglobulin G-binding protein A (Spa), which was involved in pathogenesis, was only upregulated in the evolved strain (2-fold), suggesting that it might be related to the increased elasnin tolerance phenotype. Moreover, several proteins were more downregulated in the evolved strain than in the ancestral strain, such as those involved in glycerophospholipid metabolism (glycerol-3-phosphate acyltransferase [PlsY], glycerol-3-phosphate dehydrogenase [GpsA], glycerophosphoryl diester phosphodiesterase, and CDP-diacylglycerol-glycerol-3-phosphate 3-phosphatidyltransferase, downregulated 5.6-, 12.7-, 2.1-, and 7.2-fold, respectively), and tryptophan biosynthesis (TrpC, TrpD, TrpE, downregulated 3.3-, 10.9-, and 4.2-fold, respectively), as well as endoribonuclease MazF (downregulated 2.8-fold), the toxic component of a type II toxin-antitoxin (TA) system which functions to cleaves mRNA, thereby inhibiting protein synthesis and inducing bacterial stasis. Interestingly, elasnin treatment appeared to suppress a number of proteins in the ancestral strain but had no such effect in the evolved strain. For instance, proteins for DNA binding and replication, such as anaerobic ribonucleoside-triphosphate reductase, and proteins for RNA binding and processing, such as rRNA small subunit methyltransferase H (RsmH), anti-σ^B^ factor antagonist (RsbV), and formate acetyltransferase (PflB), were downregulated 5.4-, 2.7-, 2.4-, and 2.4-fold, respectively, only in the ancestral strain and not in the evolved strain. More importantly, proteins that were involved in cell division and peptidoglycan biosynthesis, such as MurA, MurG, and ScpA, were also downregulated only in the ancestral strain (2.2-, 2.2-, and 2.9-fold, respectively).

To put these downregulated proteins in their functional context, we subjected the list of downregulated proteins in the ancestral and evolved strains to gene ontology (GO) analysis and pathway analysis by DAVID (Database for Annotation, Visualization and Integrated Discovery) (23) (Fig. 3h and i). Similar to the previous observation, we spotted that processes such as cellular iron ion homeostasis and cell wall organization were significantly downregulated in the ancestral strain. On the other hand, tryptophan biosynthesis, barrier septum assembly, and glycerophospholipid metabolism were downregulated only in the evolved strain and not in the ancestral one. Moreover, in both of the strains, processes such as transcription, purine metabolism, phosphoribosylformylglycinamidine synthase activity, and DNA and RNA binding were downregulated. Interestingly, members of the two-component system such as LytR, KdpE, VraR, WalK, and SrrB, which are known to be important for sensing the surroundings and adapting to environmental stresses, were also downregulated in both strains. Although how exactly the two-component system was suppressed upon elasnin treatment is still unknown and requires further investigation, this implies that the elasnin-treated cells are more prone to stresses, which agrees with the previous observation that they became more sensitive to β-lactams (7).

Cold shock protein CspA, which was known to regulate S. aureus pigment production (24, 25), was one of the most downregulated proteins in both the ancestral and evolved strains (∼200-fold in the ancestral strain and ∼346-fold in the evolved strain) (Table S2). Other highly downregulated proteins in both the ancestral and evolved strains were cell division proteins SepF and WhiA (downregulated 23.3- and 16.4-fold in the ancestral strain and 15.4- and 6.6-fold in the evolved strain, respectively), suggesting that cell division was slower in the presence of elasnin. It is possible that dividing and producing pigment lowered the fitness of the cells in the presence of elasnin, and hence they grew slower and lowered the expression of proteins related to pigment production, such as CspA. Upon repeated elasnin treatment such as in our evolution experiment, mutations that led to slower growth and/or abolished pigment production may have emerged and become selected.

### Transcriptome profiling of the ancestral and evolved strains under elasnin treatment.

**FIG 4:**
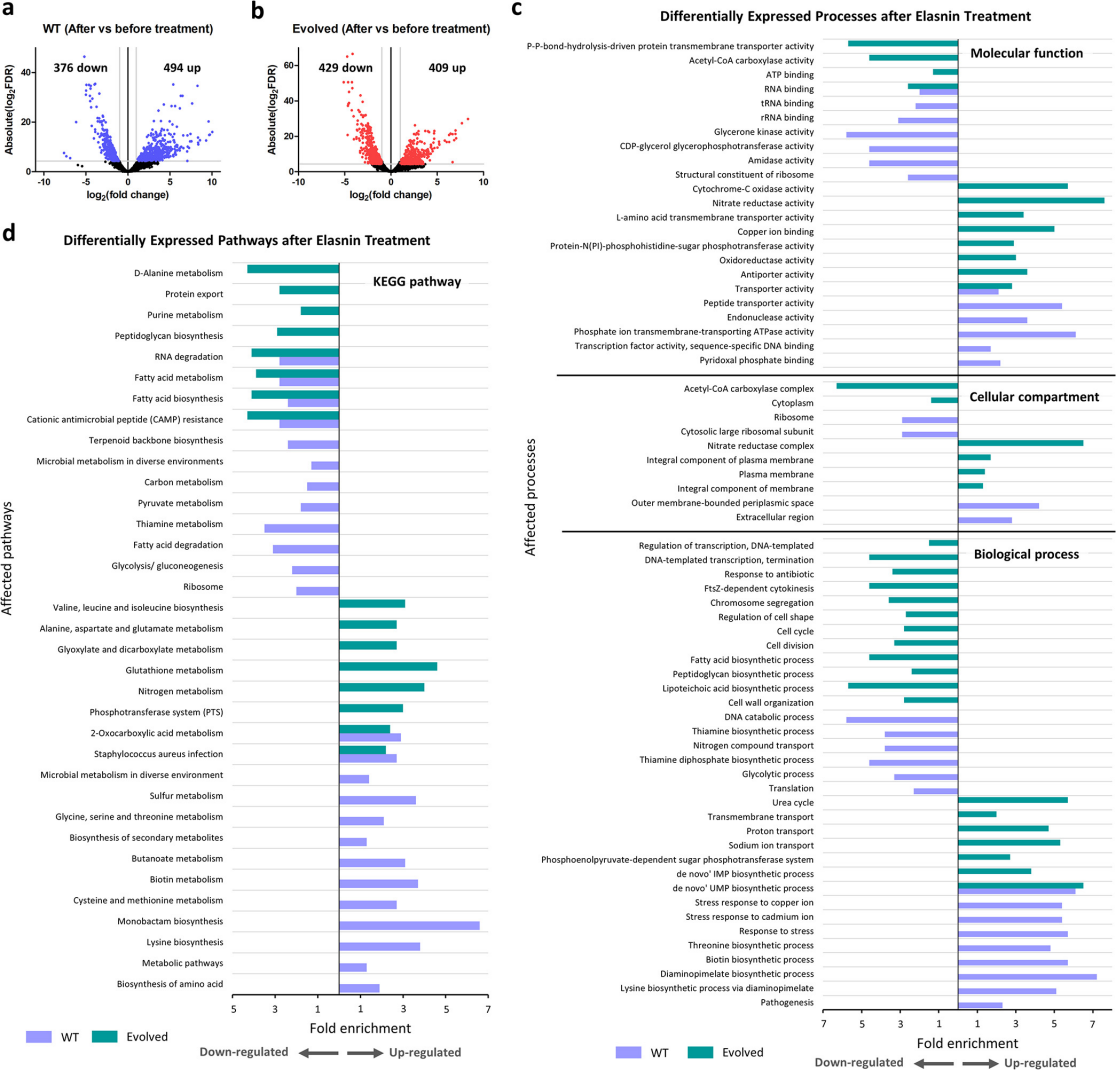
Transcriptomics analysis of the evolved and ancestral strains upon elasnin treatment. (a and b) Volcano plots of the ancestral WT (a) and the elasnin-evolved strain (b) after elasnin treatment (50 μg/mL) for 5 h compared to those before treatment. Differentially expressed genes (DEGs) were defined to be those with an FDR below 0.05, and absolute fold change greater than 2, corresponding to the colored dots. (c and d) GO analysis (c) and pathway enrichment study (KEGG) (d) by DAVID of the differentially expressed genes of ancestral WT and evolved strain after elasnin treatment. Fold enrichment was defined as the ratio of the proportion of the input information to the background information.

To complement our proteomics analysis, we performed transcriptome profiling of both the evolved and ancestral WT strains upon treatment with elasnin. The volcano plots along with the number of differentially expressed genes (DEGs), highlighting the up- and downregulated genes after elasnin treatment in both the WT and evolved strain, are shown in Fig. 4a and b, respectively. The full list of DEGs is available in Table S3. We subjected the DEGs from both the ancestral and evolved strains to GO analysis (Fig. 4c) and pathway analysis (Fig. 4d), and we observed that the two strains have quite different regulations of biological processes upon treatment with elasnin. While genes related to pathogenesis, response to stress, and biotin and lysine biosynthesis were upregulated in the ancestral strain, genes involved in the transmembrane, proton and ion transport, phosphotransferase system (PTS), and oxidative phosphorylation were upregulated in the evolved strain. Consistent with our proteomics analysis, we also observed the downregulation of genes for cell division and cell wall organization in the cells after treatment with elasnin (Fig. 4c), suggesting that elasnin interfered with the proper cell cycle, which is particularly important for growing exponential-phase cells. In addition, genes involved in RNA degradation, fatty acid biosynthesis and metabolism, and CAMP resistance were all downregulated in both strains.

10.1128/msystems.01393-21.9 TABLE S3 Differentially expressed genes (DEGs) in the ancestral and evolved strain after elasnin treatment compared those before treatment. Download Table S3, XLSX file, 0.2 MB. Copyright © 2022 Sulaiman et al. 2022 Sulaiman et al. https://creativecommons.org/licenses/by/4.0/ This content is distributed under the terms of the Creative Commons Attribution 4.0 International license.

It is interesting that the expression of *hisH* gene, which was mutated on ELAS2, was upregulated 8.3-fold in ELAS1. This gene expresses imidazole glycerol phosphate synthase subunit HisH, which is involved in the l-histidine biosynthesis pathway. Interestingly, other genes that are involved in the S. aureus histidine biosynthetic process were also upregulated upon elasnin treatment in both the ancestral and evolved strains. For instance, *hisZ*, *hisA*, *hisD*, and *hisIE* were upregulated 17.4-, 13.8-, 12.5-, and 5.2-fold in the ancestral strain, while *hisZ*, *hisF*, *hisC*, and *hisS* were upregulated 10-, 8.9-, 7-, and 2.6-fold in the evolved strain (Table S3). Therefore, beyond the mutated *hisH* gene, the overall increase in histidine biosynthesis at the pathway level seems to be important for the cell’s adaptation toward elasnin. As shown in Fig. 4d and e, not only was histidine biosynthesis upregulated but also general amino acid biosynthesis was upregulated upon elasnin treatment, such as the upregulation of lysine, valine, leucine, and isoleucine biosynthesis and also alanine, cysteine, methionine, glycine, serine, and threonine metabolism.

In contrast to our proteomics data, which revealed far fewer upregulated proteins than downregulated ones (Fig. 3e and f), the number of upregulated genes was similar to the number of downregulated genes (Fig. 4a and b). This might be due to several reasons, such as the differences in the coverage of both analyses, different filters used in specific pipelines, different sampling times, sample preparation, and the errors associated with each of these analyses. A similar poor correlation between mRNA and protein abundances has been reported by others and attributed to complicated posttranslational mechanisms and the difference in the half-lives and stability of RNA and protein, among other factors (26–30). Although the numbers of identified DEGs and DEPs were different between the proteomics and transcriptomics data sets, we observed a consistent expression pattern of the differentially expressed biological processes and pathways. First of all, the downregulated proteins have a very similar pattern to the corresponding genes. Genes/proteins involved in the two-component system (GraR, WalK, and LytR), CAMP resistance (GraR and DltD), cell shape determination (MreC), DNA binding and replication (DNA helicase, RecJ, and anaerobic ribonucleoside-triphosphate reductase), RNA binding (RsmA, RlmN, TruB, and pseudouridine synthase), transcription and regulation of transcription (NusB, Rex, MsrR, IcaR, CspA, and SarR), carbon metabolism (PflB and GpsA), purine metabolism (dITP/XTP pyrophosphatase), and transmembrane transporter activity were downregulated according to both proteomics and transcriptomics data. Most importantly, we observed that many genes/proteins for cell division (SepF, WhiA, ScpA, Sle1, and ZapA) and cell wall organization (LytM, DltD, MurG, LtaS, Sle1, autolysin SsaALP, bifunctional autolysin Atl, PBP4, IsaA, and OatA) were downregulated according to both the proteomic and transcriptomic data sets after treatment with elasnin.

**FIG 5:**
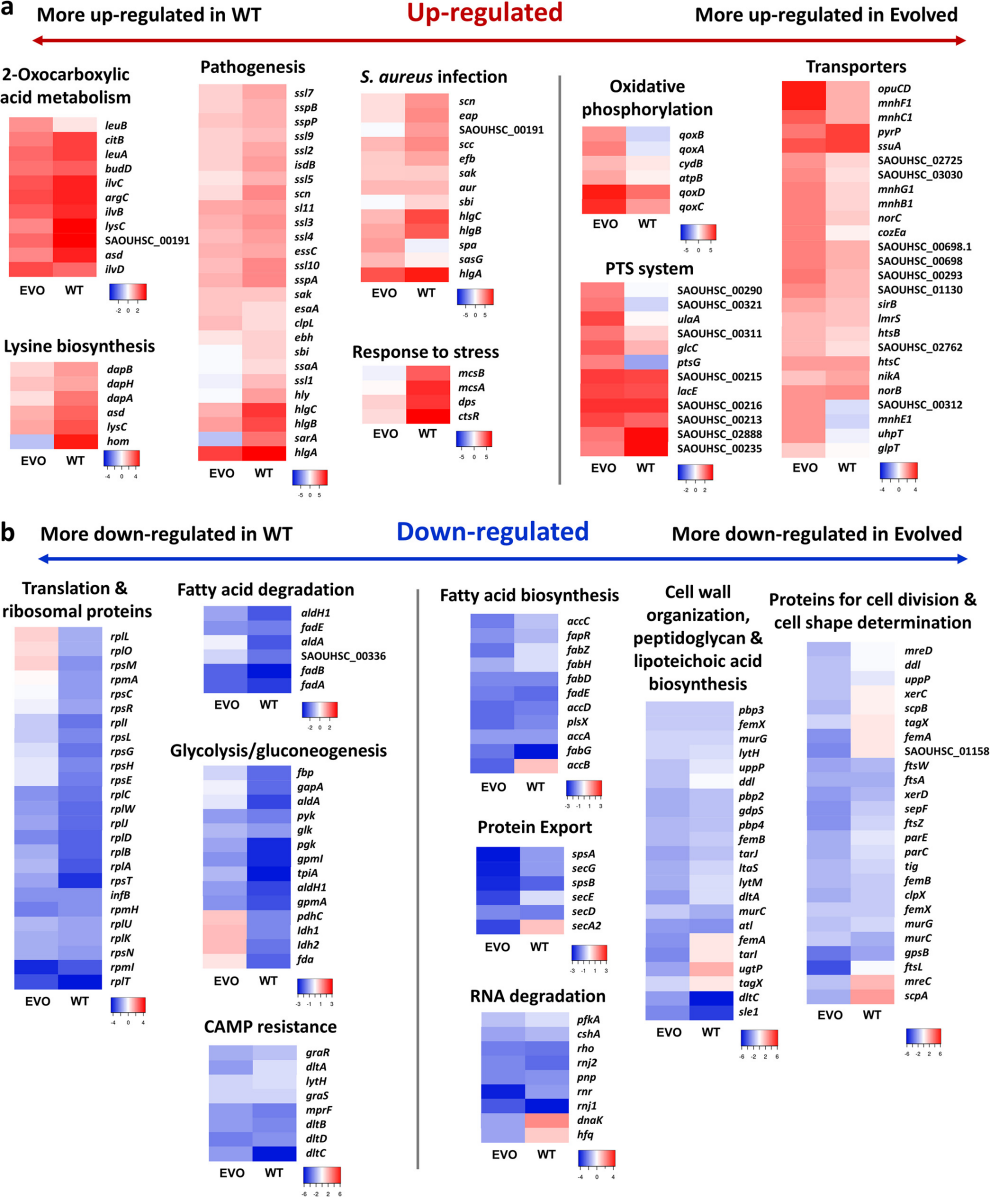
Expression pattern of selected processes in the evolved and ancestral strains upon elasnin treatment. (a and b) Heat map of log_2 (_fold change) of the upregulated (a) and downregulated (b) genes from selected cellular processes on the ancestral WT and evolved strain after elasnin treatment (compared to untreated cells). Processes on the left were more differentially expressed in the ancestral WT, and those on the right side were more differentially expressed in the evolved strain.

There was a much higher number of upregulated genes from our transcriptomics data than was the case with the upregulated proteins from the proteomics data. These were transporter genes and genes involved in oxidative phosphorylation, the PTS system, S. aureus infection and pathogenesis, response to stress, and amino acid metabolism in general, such as lysine biosynthesis and histidine biosynthesis (Fig. 4 and 5). The majority of proteins in which the genes were upregulated according to the transcriptomic data set were either not differentially expressed or not detected in the proteomic data set. Although we only detected a few upregulated proteins from proteomics, they also belong to processes similar to the ones observed from transcriptomics. For instance, transporters such as phosphate-binding protein PstS, amino acid ABC transporter SAOUHSC_02697, oligopeptide ABC transporter SAOUHSC_00926, and magnesium transport protein CorA, proteins involved in S. aureus infection and pathogenesis such as response regulator ArlR, immunoglobulin G-binding protein A (Spa), and iron-regulated surface determinant protein H (IsdH), proteins involved in oxidative phosphorylation such as probable quinol oxidase subunit 1 (QoxB), and those that play a role in lysine biosynthesis (SbnH) were all upregulated. Therefore, despite the differences in the numbers of differentially expressed genes and proteins, the proteomic data set could still capture the key alterations in the cells due to elasnin treatment.

### Different expression pattern of certain biological processes governs elasnin tolerance in the evolved strain.

We selected some differentially regulated processes from Fig. 4c and d and generated heat maps of fold changes to see the expression level of individual genes in the ancestral and evolved strains upon elasnin treatment (Fig. 5). Although in most of the processes the direction of differential expression was consistent between the ancestral and evolved strains (Fig. 5a for upregulated processes and Fig. 5b for downregulated processes), we could further categorize whether each of these processes was more differentially expressed in the ancestral or the evolved strain based on the total expression of all genes in a specific process.

Processes that were more upregulated in the ancestral strain include pathogenesis and S. aureus infection (higher expression of *sspA*, *sspB*, *sspP*, staphylococcal superantigen-like [*ssl*] genes, staphylococcal complement inhibitor gene *sci*, staphylococcal secretory antigen gene *ssaA*, immunoglobulin-binding protein gene *sbi*, transcriptional regulator gene *sarA*, and alpha-hemolysin gene *hly*), stress response (*mcsA*, *mcsB*, *dps*, and *ctsR*), lysine biosynthesis, and 2-oxocarboxylic acid metabolism, while those that were more upregulated in the evolved strain are oxidative phosphorylation (*qox* operon genes), phosphotransferase system (*ptsG*, *glcC*, and *ulaA*), and some transporter genes (including sodium ion and proton transport genes *mnhB1*, *mnhC1*, *mnhE1*, *mnhF1*, and *mnhG1*). For the downregulated processes, the ancestral strain had a much lower expression of genes involved in translation (both encoding large [*rpl*/*rpm*] and small [*rps*] ribosomal subunits), glycolysis and gluconeogenesis (*fbp*, *gapA*, *aldA*, *aldH1*, *pgk*, *gpmA*, *gpmL*, *tpiA*, *pdhC*, *ldh1*, *ldh2*, and *fda*), and cationic antimicrobial peptide resistance (lower expression of the *dlt* operon genes). On the other hand, cell wall organization was more downregulated in the evolved strain. Although most of the penicillin-binding protein genes (*pbp2*, *pbp3*, and *pbp4*) and the *mur* genes (*murC* and *murG*) had similar expression levels in the ancestral and evolved strains, the expression levels of genes involved in lipoteichoic acid biosynthesis, such as *ugtP*, *tagX*, *tarL*, and *dltA*, and some genes for peptidoglycan biosynthesis, such as *ddl* and *femA*, were much lower in the evolved strain. In addition, the expression levels of many genes involved in the cell division process, such as cell shape-determining genes *mreC* and *mreD*, cell division genes *ftsZ*, *ftsL*, and *sepF*, and segregation and condensation genes *scpA* and *scpB*, were lower in the evolved strain, consistent with the observation that the evolved strain have slower growth (Fig. 1c and d) and have trouble growing following elasnin treatment (Fig. S1b and c). The expression levels of genes involved in protein transport, including the *sec* genes (*secA2*, *secD*, *secE*, and *secG*), were also much lower in the evolved strain. This differential regulation of genes involved in specific processes might affect how the cells adapted to elasnin treatment, which finally led to the difference in elasnin sensitivity between the WT and elasnin-tolerant strains.

### The evolved strains were more sensitive toward daptomycin and lysostaphin, indicating a weaker membrane and cell wall structure.

**FIG 6:**
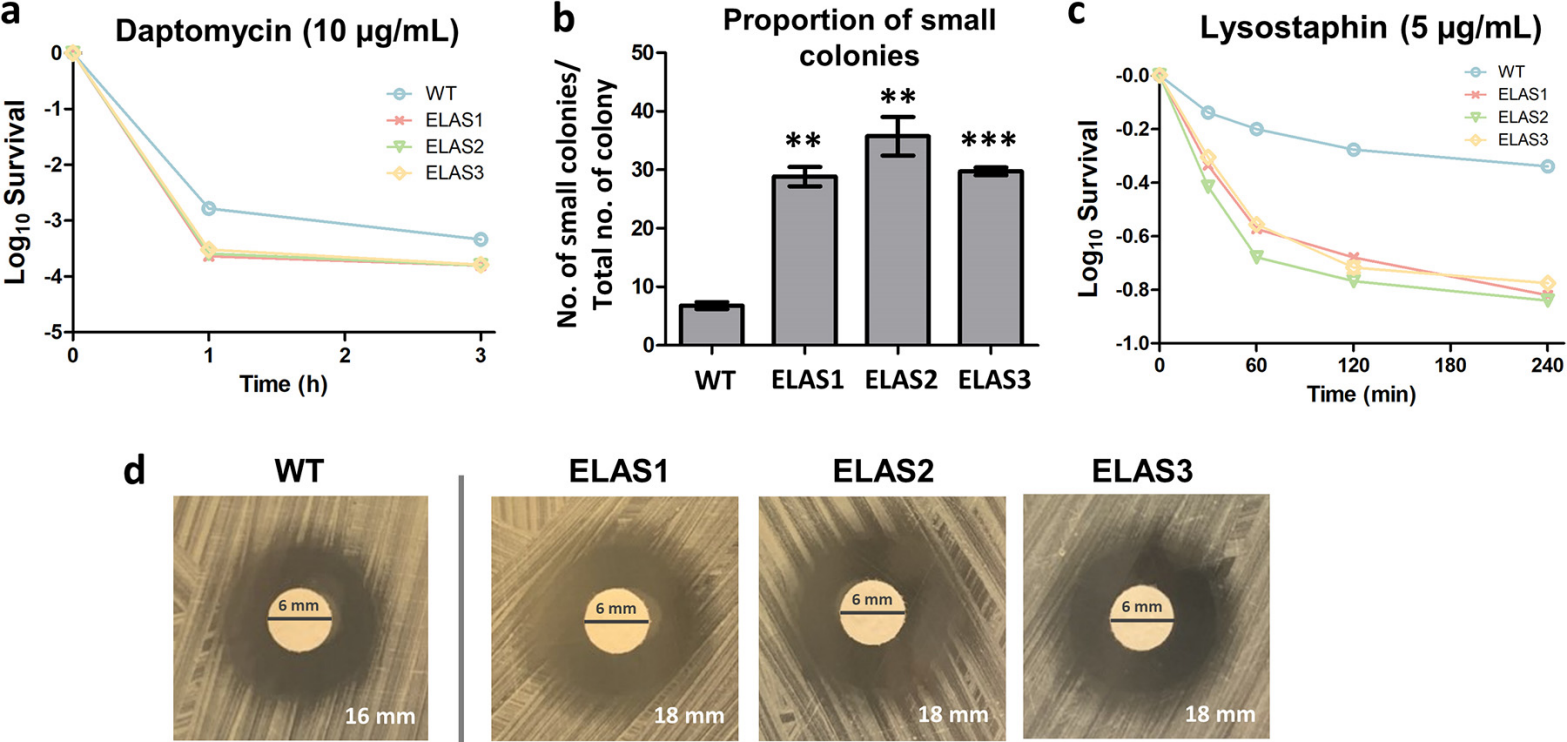
Treatment of ancestral WT and elasnin-evolved strains with daptomycin and lysostaphin. (a) Time-kill curve of exponential-phase ancestral WT and evolved strains with daptomycin. The concentration of daptomycin used was 10 μg/mL (mean ± SEM; *n* = 3). (b) Proportion of small colonies observed on the agar plate, which was defined as the number of small colonies formed divided by the total colony count in the plate (mean ± SEM; *n* = 3). **, *P* < 0.01; ***, *P* < 0.001 (two-tailed Student’s *t* test for significance of difference from the ancestral WT). (c) Lysostaphin lysis assay in the ancestral WT and evolved strains. Cells were treated with 5 μg/mL lysostaphin and incubated at 37°C, and the decrease in OD_600_ was monitored over time (mean ± SEM; *n* = 3). (d) Disc diffusion antibiotic sensitivity testing on the ancestral WT and the evolved strains toward lysostaphin (disc content, 50 μg). Images shown are representative of three replicates. The text on the lower right corner marks the diameter of the zone of inhibition.

From our proteomics and transcriptomics analyses, we observed that elasnin treatment led to significant downregulation in genes and proteins involved in cell wall organization, peptidoglycan and lipoteichoic acid biosynthesis, the two-component system, and CAMP resistance in both the ancestral and evolved strains. Since these processes have been repeatedly reported to cause resistance toward membrane- and cell wall-acting antibiotics, such as daptomycin (DAP) and vancomycin (31–34), we wondered whether our tolerant strains that were evolved from repetitive elasnin treatment were more sensitive toward these antibiotics. To test this idea, we measured the survival of the evolved strains toward the lethal dose of DAP, an antibiotic that disrupts multiple aspects of cell membrane function, and we observed that the evolved strains had lower survival upon 1 and 3 h of DAP treatment (Fig. 6a), although we observed no change in the MIC compared to that of the ancestral strain. Interestingly, we observed that most of the surviving cells from the evolved strain following DAP treatment formed small colonies when plated on agar medium (Fig. S5), with a significantly higher proportion than for the ancestral strain (Fig. 6b). This suggested that there was a significant fraction of growth-impaired cells within the evolved population due to the mutation it possessed. Moreover, when treated with lysostaphin, an endopeptidase that directly cleaves cross-linking pentaglycine bridges in the S. aureus cell wall peptidoglycan, the evolved strains showed a much lower survival (Fig. 6c). Disc diffusion assay using lysostaphin also showed that they exhibited a slight increase in the zones of inhibition compared to the ancestral WT (Fig. 6d). This indicated that they had a weaker cell wall peptidoglycan structure than that of the ancestral strain.

10.1128/msystems.01393-21.5 FIG S5 Formation of small colonies on the evolved strains upon treatment with daptomycin (10 μg/mL) for 3 h. Cells were washed, serially diluted, and plated on MH agar (21-h incubation time). White arrows indicate colonies with sizes at least 2.23 times smaller in radius than the normal-size colonies, a general criterion used to identify small-colony variants (SCVs). The images are representative images from 3 biological replicates. Download FIG S5, JPG file, 0.5 MB. Copyright © 2022 Sulaiman et al. 2022 Sulaiman et al. https://creativecommons.org/licenses/by/4.0/ This content is distributed under the terms of the Creative Commons Attribution 4.0 International license.

## DISCUSSION

Elasnin was recently reported to exhibit antibiofilm activity against multiple Gram-positive and -negative bacteria and can be used as a coating agent in marine environments (5). Furthermore, a mechanistic study of its antibiofilm activity in MRSA has been recently performed, revealing that elasnin suppressed the production of virulence factors which resulted in a lower expression of extracellular EPS, cell wall, and membrane components, as well as peptidoglycan hydrolases, which are important for cell wall formation during cell division (7). Subsequently, the biofilm matrix was destroyed, leading to the release of cell wall-defective biofilm cells that were more sensitive to β-lactam antibiotics. Following up on those promising results, we sought to further evaluate the potential of elasnin as an antibiotic or a codrug to another antibiotic in this study. In this study, we found that although elasnin can barely kill nongrowing stationary-phase cells upon prolonged treatment, it exhibited antibacterial activity against growing S. aureus cells in planktonic culture, albeit with slow killing kinetics (Fig. S1a). This suggested that elasnin has active targets in S. aureus planktonic cells. We also showed that upon a single elasnin treatment on WT planktonic cells, they formed a significant amount of small-colony variants (SCVs), indicating that cells surviving elasnin treatment might have trouble growing and replicating (Fig. S1b and c). Through adaptive laboratory evolution (ALE) experiments, we generated three evolved strains with increased elasnin tolerance which was accompanied by a reduced growth rate, reinforcing the idea that elasnin targets active processes (Fig. 1).

Whole-genome sequencing revealed that the evolved strains have a common single point mutation in a putative phosphate transport regulator, indicating that the intracellular phosphate may play a crucial role in elasnin tolerance (Table S1). Inhibition of this gene in the WT changed its phenotype to one resembling those observed in the evolved strains, which were increased elasnin tolerance, reduced growth rate, and loss of pigmentation (Fig. 1e and f and Fig. S2e and S3). Measurement of the intracellular phosphate (P_i_) and polyphosphate (polyP) revealed that the evolved strains had a significantly higher level of both intracellular P_i_ and polyP than the ancestral WT strain (Fig. 2). The gene *pstS*, expressing phosphate-binding protein PstS, part of the ABC transporter complex PstSACB (involved in phosphate transport), was upregulated 332- and 3.5-fold upon elasnin treatment in the wild-type and evolved strains, respectively (Table S3), whereas the protein PstS was also observed to be upregulated in the evolved strain 3.1-fold upon elasnin treatment (Table S2). A similar phenomenon has been previously reported, in which a daptomycin-tolerant S. aureus strain from repetitive DAP treatment possessed a mutation in the *pitA6* gene (expressing inorganic phosphate transporter) and had increased P_i_ and polyP level compared to the ancestral WT (35). In a follow-up study, it was concluded that the increased polyP level did not contribute to the daptomycin tolerance phenotype, but rather, the elevated P_i_ concentration directly or indirectly promoted the upregulation of the *dlt* operon, which is, in turn, responsible for DAP tolerance (36). In our case, since our evolved strains did not have increased tolerance toward DAP (but rather had decreased tolerance [Fig. 6a]), and our transcriptomics analysis showed the downregulation of the *dlt* operon genes instead (Fig. 5b), the cause-and-effect relationship between the increased level of P_i_ and polyP and elasnin tolerance may be distinct from what was reported for their daptomycin-tolerant strain. Although we currently do not know how exactly the increased P_i_, increased polyP, or both affect elasnin tolerance phenotype, some connections between the increase in P_i_ levels and S. aureus tolerance toward other membrane-acting cationic antimicrobial peptides have been previously suggested (35, 37–40). On the other hand, polyP is pivotal in modulating various stress-related networks in bacteria (41), and a fatty acid signaling molecule (*cis*-2-decenoic acid) that was known to make drug-tolerant persister cells more drug sensitive favors the enzymatic degradation of polyP (42). Therefore, the increased polyP levels in our elasnin-evolved strains might also be related to their increased tolerance phenotype.

Through proteomic and transcriptomic analysis, we observed that in general, elasnin led to the downregulation of many genes and proteins involved in cell wall organization and cell division, supporting the hypothesis that elasnin interfered with cell proliferation, which is particularly important for exponential-phase cells. This might explain why it exhibited antibacterial activity against the exponential-phase cells but could not kill stationary-phase cells (Fig. S1a) or biofilm cells (7). Other downregulated proteins were ribosomal proteins, those involved in glycolysis and gluconeogenesis, CAMP resistance, the two-component system, purine metabolism, and fatty acid metabolism, and the upregulated processes were pathogenesis and infection, the PTS system, transporters, lysine biosynthesis, response to stress, and oxidative phosphorylation (Fig. 3 and 4). Based on the overall expression of the genes in each of these processes, the process could then be categorized as more differentially expressed in the evolved strain or the WT strain. For instance, pathogenesis and infection, stress response, lysine biosynthesis, and 2-oxocarboxylic acid metabolism were more upregulated in the ancestral strain, whereas oxidative phosphorylation, the PTS system, and some transporter genes were more upregulated in the evolved strain (Fig. 5a). Moreover, while ribosomal proteins, glycolysis, gluconeogenesis, and CAMP resistance were more downregulated in the ancestral strain, cell wall organization, cell division, and protein transport were more downregulated in the evolved strain (Fig. 5b). This differential expression of specific processes could be related to their difference in susceptibility toward elasnin.

We observed that the evolved strains that were subjected to repetitive elasnin treatment lost their characteristic orange pigment (Fig. S2a to d), which means that the cells favored limited pigment synthesis in the presence of elasnin. From our transcriptomics data, we observed that two genes that were involved in staphyloxanthin production (see Fig. S6 for the diagram of staphyloxanthin biosynthesis), *crtO* (glycosyl-4,4′-diaponeurosporenoate acyltransferase) and *aldH* (4,4′-diaponeurosporen-aldehyde dehydrogenase), were both downregulated 5.6-fold in the ancestral WT strain upon elasnin treatment (Table S3). AldH catalyzes the oxidation of 4,4′-diaponeurosporen-4-al to yield 4,4′-diaponeurosporenoic acid, while CrtO catalyzes the acylation of glycosyl-4,4′-diaponeurosporenoic acid to yield staphyloxanthin, which is the last step in the biosynthesis of this orange pigment. Therefore, the downregulation of these two enzymes would lead to lower staphyloxanthin pigment production upon elasnin treatment. In addition, protein CspA, which is known to regulate S. aureus pigment production through a σ^B^-dependent manner (24, 25), was downregulated 200-fold and 346-fold in the ancestral WT and evolved strain upon elasnin treatment. This observation was further reinforced by the downregulation of the protein anti-σ^B^ factor antagonist (RsbV), which is the positive regulator of σ^B^ activity (downregulated 2.4-fold in protein level and 3.7-fold in gene expression level in the ancestral strain upon elasnin treatment) (Tables S2 and S3). Some other upstream factors, such as *spx* and *clpX* (25), were also found to be differentially regulated. In conclusion, elasnin treatment caused a reduction in staphyloxanthin production, which may lead to lower tolerance to oxidative stress given that the pigment acts as a scavenger of free radicals (20), though this requires further investigation.

10.1128/msystems.01393-21.6 FIG S6 Diagram for staphyloxanthin biosynthesis in S. aureus. The expression of *aldH* and *crtO* genes was downregulated 5.6-fold in the ancestral WT upon elasnin treatment. Upstream of the staphyloxanthin biosynthetic process that was regulated through a σ^B^-dependent manner, the expression of CspA protein was downregulated 200-fold in the ancestral WT upon elasnin treatment. Some other upstream factors, such as *spx* and *clpX*, were also found to be differentially regulated. Download FIG S6, TIF file, 0.9 MB. Copyright © 2022 Sulaiman et al. 2022 Sulaiman et al. https://creativecommons.org/licenses/by/4.0/ This content is distributed under the terms of the Creative Commons Attribution 4.0 International license.

Our findings pointed out the potential clinical applications of elasnin. Besides its biofilm eradication activity toward MRSA (7), it can also kill the growing planktonic cells. It was reported previously that biofilm cells released upon elasnin treatment have increased susceptibility toward β-lactams (7), and in this study, we further discovered that elasnin-evolved strains isolated from populations subjected to repetitive elasnin treatment became more sensitive toward daptomycin and lysostaphin (Fig. 6). Therefore, combining elasnin with other membrane- or cell wall-acting antibiotics in a combinatory treatment may be an effective strategy to treat S. aureus infections. Overall, through transcriptomic and proteomic analyses, we revealed key processes affected by elasnin in growing S. aureus cells and gained insights into the biological changes associated with elasnin tolerance.

## MATERIALS AND METHODS

### Bacterial strains and growth conditions.

The bacterial strain used in this study was methicillin-resistant S. aureus (MRSA) ATCC 43300. Exponential-phase cultures were prepared by incubating a 1:1,000-diluted overnight culture in cation-adjusted Mueller-Hinton (MH) broth (supplemented with 50 mg/L Ca^2+^) until the optical density at 600 nm (OD_600_) reached ∼0.1 at 37°C with shaking. MH agar was used for colony counts.

### Preparation of elasnin.

Elasnin was prepared according to a previously described protocol (5). Briefly, stock cultures of Streptomyces mobaraensis DSM 40847 were inoculated into AM4 medium (soybean powder [20 g/L], bacteriological peptone [2 g/L], glucose [20 g/L], soluble starch [5 g/L], yeast extract [2 g/L], NaCl [4 g/L], K_2_HPO_4_ [0.5 g/L], MgSO_4_·7H_2_O [0.5 g/L], CaCO_3_ [2 g/L] [pH 7.8]) and incubated at 30°C for 5 days. The culture broth was then extracted with ethyl acetate, and pure elasnin was isolated by reversed-phase high-performance liquid chromatography (HPLC) (2695; Waters, Milford, MA) using a semiprep C_18_ column (10 by 250 mm) and dissolved in dimethyl sulfoxide (DMSO) before storage and bioassay.

### Evolution experiment.

Exponential-phase wild-type MRSA culture was exposed to a lethal dose of elasnin (50 μg/mL) for 5 h. The antibiotic-containing medium was removed by washing three times in MH broth (10 min of centrifugation at 4,500 × *g*), and the cells were resuspended in 1 mL fresh MH broth and grown overnight at 37°C with shaking. The cycle was repeated 7 times (1 week). Three independent evolution experiments were performed on WT MRSA, generating three evolved strains, ELAS1, ELAS2, and ELAS3.

### Tolerance and resistance assay.

The concentration of elasnin used for treatment was 50 μg/mL, and the concentration of daptomycin used for treatment was 10 μg/mL. To assess cell viability after antibiotic treatment, the survivors were counted by serially diluting cultures in MH broth, plating 100 μL on MH agar and spread plates. The MICs of the population were recorded by the broth macrodilution method. Briefly, the MIC was determined by incubating ∼5 × 10^5^ exponential-phase bacteria in MH medium overnight with various concentrations of antibiotics. The MIC value was determined as the lowest concentration without growth, according to EUCAST guidelines.

Disc diffusion assay was performed to see the sensitivity of the evolved strains toward lysostaphin, since MIC measurements by broth macrodilution might not be appropriate as previously discussed (43). The disc diffusion test was performed according to the standard EUCAST susceptibility test guideline in which inoculum suspension equivalent to a 0.5 McFarland standard (1 × 10^8^ to 2 × 10^8^ CFU/mL) was spread on MH agar applied with an antimicrobial disk containing 50 μg lysostaphin and incubated at 35°C for 20 h.

### Genomic extraction and whole-genome sequencing.

The genomic DNA from ancestral and evolved strains was extracted using the DNeasy blood and tissue kit (Qiagen) according to the manufacturer’s protocol, with the following lysis buffer: 200 μg/mL lysostaphin solution in 20 mM Tris-HCl (pH 8.0), 2 mM sodium EDTA, and 1.2% Triton X-100. DNA was detected by the agarose gel electrophoresis and quantified by a NanoVue Plus spectrophotometer (GE Healthcare). The genomic DNA was sent to BGI for paired-end DNBseq sequencing at 2 × 150-bp read length and 350-bp insert size. Briefly, a total amount of 1 μg DNA sample was used as input material for the DNA sample preparation. The DNA sample was fragmented by ultrasound on a Covaris E220 ultrasonicator (Covaris, Brighton, UK) and selected with an Agencourt AMPure XP-Medium kit to an average size of 200 to 400 bp. DNA fragments were end repaired, 3′ adenylated, and ligated with the adapter. The ligation product with adapters was then amplified, and PCR products were purified with the Agencourt AMPure XP-Medium kit. The double-stranded PCR products were then heat denatured and circularized by the splint oligonucleotide sequence. The remaining linear molecule was digested with exonuclease. The single-strand circle DNA (ssCir DNA) was formatted as the final library and sequenced using BGISEQ-500 where the ssCir DNA molecule formed a DNA nanoball (DNB) (44) containing more than 300 copies through rolling-cycle replication. The DNBs were loaded into the patterned nanoarray by using high-density DNA nanochip technology. Finally, pair-end 150-bp reads were obtained by combinatorial probe-anchor synthesis (cPAS). Sequencing quality was affirmed using the FastQC algorithm. The sequenced data were filtered, and adapter sequence and low-quality data were removed, resulting in the clean data used for subsequent analysis. Specific processing steps were as follows: removal of reads whose low-quality nucleotides (Q value ≤ 12) exceeded a certain threshold (50% by default), elimination of reads which contained N nucleotides exceeding a certain threshold (50% by default), elimination of adapter contamination, and, finally, filtering of the duplication. The clean bases of each sample are ∼1.3 billion bp and the clean reads are ∼8.7 million reads for each sample. The whole-genome sequencing raw data are accessible under BioProject no. PRJNA699400.

### Whole-genome sequencing data analysis.

We performed a genomic comparison between the ancestral and evolved strains to the reference genome. The variation information of the sample and the reference was obtained by aligning the sample reads with the reference genome (MRSA ATCC 43300 genome downloaded from the ATCC website, September 2020) using BWA mapper v0.7.17 (45). The parameters of BWA are as follows: mem-t 4 -k 32 -M-R. The mapping rate was above 99.13% for all strains. SAMTOOLS v1.9 (46) was used to detect single nucleotide polymorphisms (SNPs) and small indels (<50 bp) with the following parameters: mpileup-m 2 -F 0.002 -d 10000 -u-L 10000 and call --ploidy 1 -mv-Ov. The detected SNPs were further filtered with QUAL of  >50. Therefore, the final SNP list contained high-quality SNPs with high confidence. Subsequently, Integrative Genomics Viewer (IGV) (47) was used to view the aligned sequence and perform further analysis on the identified SNPs/indels (e.g., determination of amino acid substitution). To verify our results, Snippy V4.6.0 (48), a rapid haploid variant calling and core genome alignment software that has a built-in filter to detect high-quality SNPs, was used to reanalyze the whole-genome sequencing data. No difference was found in the identification of SNPs using SAMTOOLS or Snippy.

### Pigment quantification.

To extract the staphyloxanthin pigment and other intermediate carotenoids, wild-type ancestral and evolved/mutant strains were grown at 37°C for 24 h. Cells were centrifuged and washed twice with phosphate-buffered saline (PBS). An equal amount of cells was resuspended in 1,000 μL methanol and heated at 55°C for 1 h with shaking. The methanol extract was subsequently cooled and centrifuged, and the supernatant was taken. Pigment content was quantified spectrophotometrically by measuring the absorbance spectrum of the methanol extract and also by measuring the OD_450_ (49).

### Lysostaphin lysis assay.

In addition to the disc diffusion assay to see the sensitivity of the evolved strains toward lysostaphin, we also performed a lysostaphin lysis assay, or turbidity assay (43). The lysostaphin lysis assay was performed following protocols described in the literature, with a slight modification (50, 51). Cells were grown to an OD_600_ of ∼0.6 and harvested by centrifugation. Cells were washed with water and resuspended in PBS supplemented with 5 μg/mL lysostaphin (Sigma-Aldrich). Cells were then incubated at 37°C, and the decrease in OD_600_ was monitored over time.

### Measurement of inorganic phosphate concentration.

A commercially available kit (phosphate assay kit, ab65622; Abcam) was used to quantify intracellular phosphate (P_i_) levels, following a similar previously described protocol for S. aureus (35). The ancestral and evolved strains were inoculated in MH broth for 16 h at 37°C with shaking. The cultures were chilled on ice for 15 min and centrifuged, and the cell pellets were washed twice in double-distilled water and adjusted to the same OD_600_ (0.5) in double-distilled water. The cells were sonicated for 3 min each and centrifuged, and the supernatants were used to determine the P_i_ levels according to the manufacturer’s instructions. A standard curve was prepared to calculate the actual P_i_ concentration using a 10 mM phosphate standard (Abcam).

### Determination of the relative abundance of polyphosphate.

Intracellular polyphosphate (polyP) levels were determined using 4′,6-diamidino-2-phenylindole (DAPI), as described previously (35, 52, 53). The ancestral and evolved strains were grown in MH broth for 16 h at 37°C with shaking. Cells were washed twice and resuspended in Tris-HCl buffer (100 mM Tris [pH 7.5]). All cell suspensions were adjusted to the same OD_600_ (0.5), and DAPI was added to a final concentration of 20 μM. After 30 min of agitation at 37°C, fluorescence was determined in a 96-well plate using a microplate reader. DAPI-polyP complexes were excited with a 415-nm laser and emission at 550 nm was recorded, whereas DAPI bound to DNA was excited with a 358-nm laser and emission at 461 nm was recorded. At the 550-nm wavelength, where the fluorescence of DAPI-polyP was measured, the emission signals of free DAPI and DAPI bound to DNA were minimal (53). Confocal images of strains stained with DAPI (final concentration, 10 μM) were obtained with a Zeiss LSM-710 confocal microscope, using a 63×/1.40 oil immersion objective and Zen software.

### Sample preparation for proteomics.

The ancestral WT and elasnin-tolerant evolved strain were grown to exponential phase and treated with elasnin (50 μg/mL) for 5 h. Untreated ancestral and evolved populations were collected as a control. For all samples, three biological replicates including the control samples were performed.

We adopted a previously described sample preparation workflow that maximize protein identification on S. aureus samples, including the extraction of surface-associated proteins (54). The cell pellet was suspended in 350 μL lysis buffer (8 M urea, 50 mM Tris-HCl [pH 8.0]), frozen in liquid nitrogen, and sonicated for 15 min. The sample was centrifuged (16,000 × *g* for 10 min) to remove cell debris and insoluble materials. An aliquot of the sample was taken for bicinchoninic acid (BCA) protein assay (Pierce BCA protein assay kit). After protein quantification, the sample was reduced with dithiothreitol (DTT; 0.1 M final concentration) at 37°C for 1 h. For shotgun proteomics, 100 μg proteins was mixed with up to 250 μL exchange buffer (6 M urea, 50 mM Tris-HCl [pH 8.0], 600 mM guanidine HCl), transferred to an Amicon filter device (Millipore, Darmstadt, Germany), and centrifuged (14,000 × *g* for 20 min). The proteins in the filter device were alkylated with iodoacetamide (IAA; 50 mM in exchange buffer) in the dark for 20 min and then centrifuged (14,000 × *g* for 20 min). To dilute the urea concentration, 250 μL 50 mM ammonium bicarbonate was added to the filter device and centrifuged (14,000 × *g* for 20 min). This step was repeated once. Proteins were digested with sequencing-grade modified trypsin (1:50 [wt/wt]; Promega, Madison, WI) for 12 h at 37°C. Then the sample was acidified with 10% formic acid to a final concentration of 0.1% (vol/vol) and centrifuged at 16,000 × *g* for 5 min. Finally, the samples were desalted by C_18_ reverse-phase ZipTip chromatography (Millipore, Darmstadt, Germany) and dried with SpeedVac (Eppendorf, Hamburg, Germany) for 20 min.

### Liquid chromatography.

The samples were reconstituted in 25 μL water-acetonitrile-formic acid in a 97.9:2:0.1 (vol/vol/vol) ratio and processed through a Bruker nanoElute ultrahigh-performance liquid chromatograph (UHPLC; Bruker Daltonics, Bremen, Germany) coupled to a hybrid trapped-ion mobility-quadrupole time-of-flight mass spectrometer (TimsTOF Pro, Bruker Daltonics, Bremen, Germany) via a nanoelectrospray ion source (CaptiveSpray; Bruker Daltonics). A volume of 1 μL (approximately 200 ng protein digest) was injected into the UHPLC system and separated on an IonOpticks 25-cm Aurora Series Emitter column with CaptiveSpray insert (250 mm by 75-μm internal diameter, 120-Å pore size, 1.6-μm particle size C_18_) at a flow rate of 0.3 μL/min. The mobile phase composition was 0.1% formic acid in water for solvent A and 0.1% formic acid in acetonitrile for solvent B. The gradient was applied from 2% to 5% solvent B for 0.5 min, from 5% to 30% solvent B for 26.5 min, and then from 30% to 95% solvent B for 0.5 min. In the end, the mobile phase was kept at 95% solvent B for 0.5 min and then decreased to 2% solvent B for 0.1 min. Two minutes of equilibration with 2% solvent B was applied before the next injection.

### TimsTOF Pro mass spectrometer.

A detailed description of the mass spectrometer can be found in previous reports (55, 56). Briefly, ions from the CaptiveSpray ion source enter the first vacuum stage, where they are deflected by 90° and accumulated in the front part of a dual trapped-ion mobility spectrometry (TIMS) analyzer. An RF potential of 300 V_pp_ is applied to radially trap the ion cloud. After the initial accumulation step, ions are transferred to the second region of the TIMS analyzer to perform ion mobility analysis in parallel. In both parts of TIMS analyzer, the RF voltage is superimposed by an increasing longitudinal electrical field gradient, such that ions in the tunnel are dragged by the incoming gas flow from the source and repulsed by the electrical field at the same time. Ramping down the electrical field releases ions from the TIMS analyzer in order of their ion mobility for quadrupole time of flight (QTOF) mass analysis. The dual TIMS setup enables operation at 100% duty cycle, when accumulation and ramp times are kept equal. In this study, we set the accumulation and ramp time to 100 ms each and recorded mass spectra in the range from *m/z* 100 to 1,700 using the positive electrospray mode. The ion mobility was scanned from 0.85 to 1.30 V∗s∗cm^−2^. The quadrupole isolation width was set to 2 Th for *m/z* <700 and 3 Th for *m/z* >700, and the collision energy was linearly increased from 27 eV to 45 eV as a function of increasing ion mobility. The overall acquisition cycle of 0.53 s comprised one full TIMS-MS scan and four parallel accumulation-serial fragmentation (PASEF) tandem mass spectrometry (MS/MS) scans. Low-abundance precursor ions with an intensity above a threshold of 2,500 counts but below a target value of 20,000 counts were repeatedly scheduled and otherwise dynamically excluded for 0.4 min. The TIMS dimension was calibrated linearly using three selected ions from the Agilent electrospray ionization (ESI) LC-MS tuning mix (*m/z*, 1/K0: [622.0289, 0.9848 Vs cm^−2^], [922.0097, 1.1895 Vs cm^−2^], [1221,9906, 1.3820 Vs cm^−2^]) in positive mode.

### Database searching and LFQ of proteomics data.

The raw data were processed together in a single run by MaxQuant v1.6.17.0 (57, 58) with default parameters except the following: “discard unmodified counterpart peptides” was set to false, “write msScans table” was set to true, and “match between runs” was set to true. For label-free quantification (LFQ), the minimum ratio count was set to 2, and separate LFQ in parameter groups was set to true. Database searches were performed using the Andromeda search engine, which was integrated in MaxQuant (59), using a custom database. Briefly, the genome sequence of MRSA ATCC 43300 was converted into a protein database using the GeneMark (60) (v3.25) gene prediction tool. The proteins were then annotated using BLASTp (v2.7.1) from NCBI using MRSA NCTC 8325 as the protein database. The sequences of common contaminants, such as trypsin and human keratins, were added to the database.

Data processing was performed using Perseus v1.6.15.0 (61). MaxQuant employs the MaxLFQ algorithm for LFQ, and quantification was performed using both unique peptides and razor peptides (peptides that match multiple protein groups, which are assigned to the protein group with the most unique peptides), including those modified by acetylation (protein N terminus) and oxidation (Met). Briefly, protein group LFQ intensities were log_2_ transformed to reduce the effect of outliers. To overcome the obstacle of missing LFQ values, missing values were imputed from the normal distribution. Log ratios were calculated as the difference in the average log_2_ LFQ intensity values between experimental and control groups. Two-tailed Student’s *t* test calculations were used in statistical tests. A protein was considered differentially expressed if its fold change was higher or lower than ±2-fold and the permutation-based false discovery rate (FDR) was ≤0.05. All the differentially expressed proteins were used in the subsequent protein network and pathway analysis.

### Bioinformatics analysis for proteomics.

We visualize our proteomic data using principal-component analysis (PCA) of the log_2_ LFQ intensity values using Perseus. To compare the protein expression profiles between different populations, we generated a heat map of fold changes of the differentially expressed proteins across the ancestral and evolved strains. DAVID (Database for Annotation, Visualization and Integrated Discovery) v6.8 (23) was used for gene ontology (GO) and pathway analyses.

### Transcriptome analysis.

Cells for transcriptome analysis were collected under the same growth and treatment conditions as those for proteomics analysis. Briefly, the ancestral WT and elasnin-tolerant evolved strain were grown to exponential phase and treated with elasnin (50 μg/mL) for 5 h. Untreated ancestral and evolved populations were also collected as controls. For all samples, three biological replicates including the control samples were performed.

Upon the collection of cells, RNA was immediately stabilized with RNAprotect bacterial reagent (Qiagen, Hilden, Germany) according to the manufacturer’s protocol. Total RNA was then extracted with an RNeasy PowerBiofilm kit (Qiagen) and sequenced using the Illumina NovaSeq platform with the paired-end mode and read length of 150 bp. Approximately 2 Gb of raw reads was generated for each sample. The raw reads were cleaned (removal of low-quality and adapter sequences) with Trimmomatic v0.36 (62) and then mapped to the S. aureus ATCC 43300 genome (retrieved from the ATCC website on January 2021) via Bowtie2 v2.3.5 (63), followed by transcript quantification using Salmon v0.13.1 (64). Differentially expressed genes were analyzed using edgeR with scripts “align_and_estimate_abundance.pl” and “run_DE_analysis.pl” implemented in Trinity v2.8.5 (65, 66). Genes with an FDR below 0.05 and fold change lower or higher than two were considered differentially expressed genes (DEGs).

### Transcription inhibition of the mutated gene.

The expression inhibition of the putative phosphate transport regulator (the mutated gene observed in the evolve strains) was accomplished using CRISPR/Cas9 system pCasiSA. The bacterial strains, plasmids, and primers used in this study are listed in Table S4. Plasmid pCasiSA was constructed by modifying the pCasiSA plasmid obtained from Quanjiang Ji as previously described (21) (Addgene; plasmid 98211; http://n2t.net/addgene:98211; RRID, Addgene_98211). Briefly, competent cells were first prepared as previously described and stored at −80°C (21). Then, the constructed plasmid was electroporated into the wild-type MRSA ATCC 43300 strain by thawing 50 μL competent cells on ice for 10 min, mixing them with 1 to 2 μg plasmid, and transferring them into a 1-mm electroporation cuvette (Bio-Rad, Hercules, CA). Cells were pulsed at 2.5 kV, 100 Ω, and 25 μF and incubated in 1 mL tryptic soy broth (TSB) at 30°C for 1 h, followed by plating on a TSB agar plate containing 7.5 μg/mL chloramphenicol (CHL) for screening. The plates were incubated overnight at 30°C. Mutant strains were then subjected to relevant tolerance and resistance assays, growth rate measurement, and pigment quantification. Cells were grown at 30°C for all assays involving the mutant strains.

10.1128/msystems.01393-21.10 TABLE S4 Bacterial strains, plasmids, and primers used in this study. Download Table S4, DOCX file, 0.01 MB. Copyright © 2022 Sulaiman et al. 2022 Sulaiman et al. https://creativecommons.org/licenses/by/4.0/ This content is distributed under the terms of the Creative Commons Attribution 4.0 International license.

### Data availability.

Whole-genome sequence data have been deposited in the BioProject database under the accession number PRJNA699400. The mass spectrometry proteomics data have been deposited to ProteomeXchange via the PRIDE repository with the data set identifier PXD024005. The raw RNA sequencing data have been deposited to the National Center for Biotechnology Information’s Sequence Read Archive database with the BioProject number PRJNA741084.
