# Supplementary figures and images for: Proteomics and Transcriptomics Uncover Key Processes for Elasnin Tolerance in Methicillin-Resistant Staphylococcus aureus
Source: mSystems. 2022 Jan 25;7(1):e01393-21. doi: 10.1128/msystems.01393-21 (PMC8788329; doi:10.1128/msystems.01393-21)

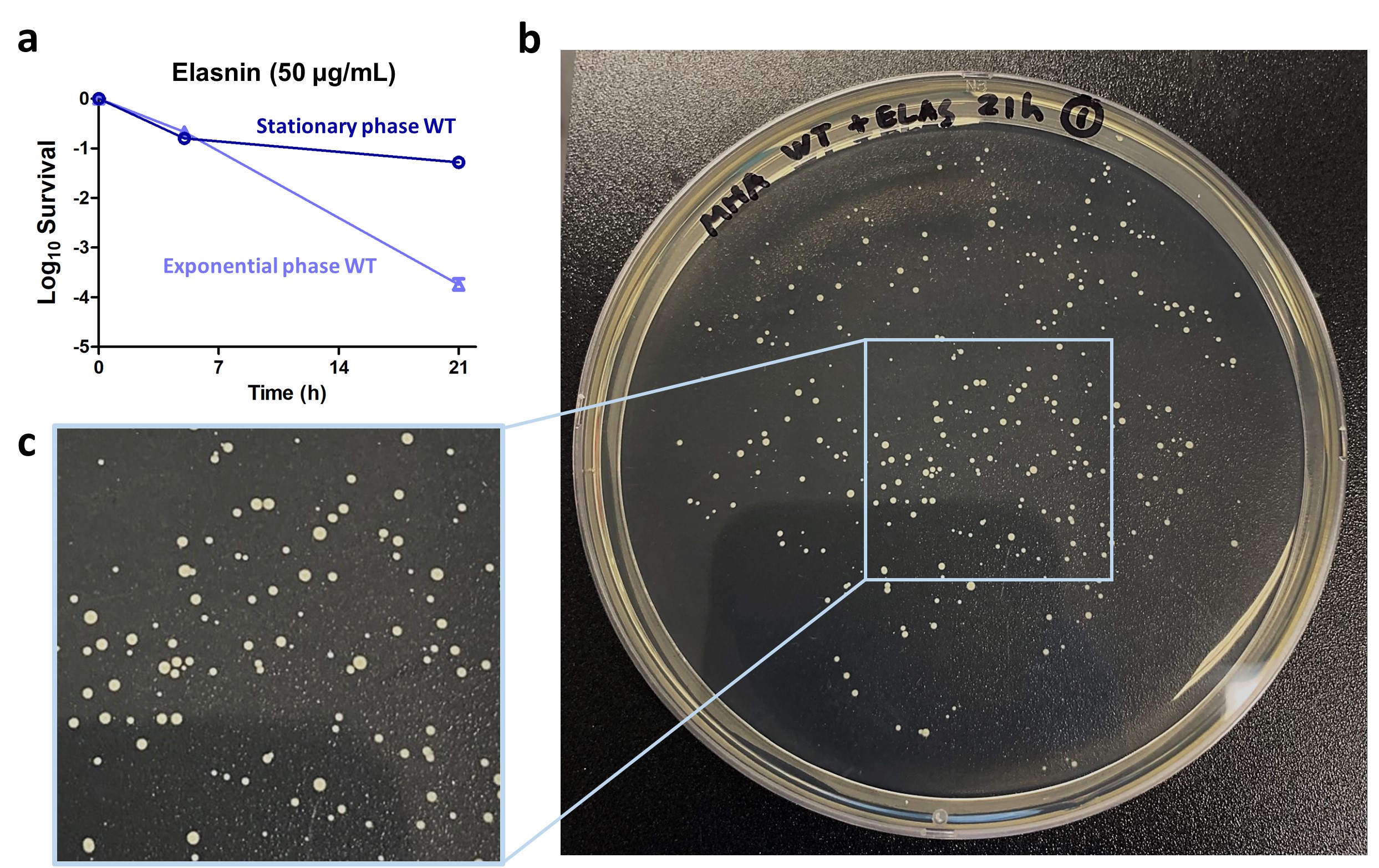

Supplement: FIG S1 [file msystems.01393-21-sf001.jpg]

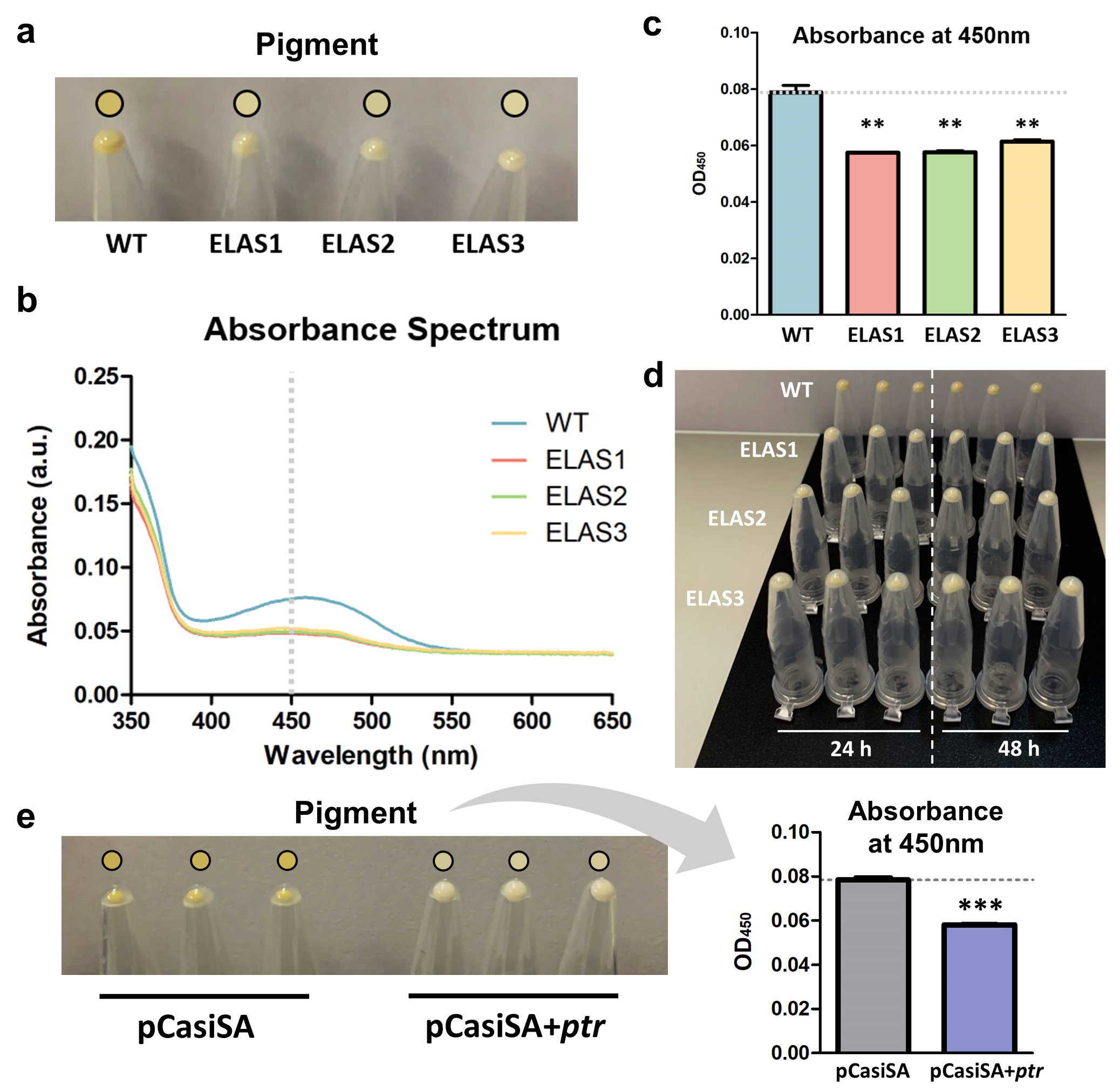

Supplement: FIG S2 [file msystems.01393-21-sf002.tif]

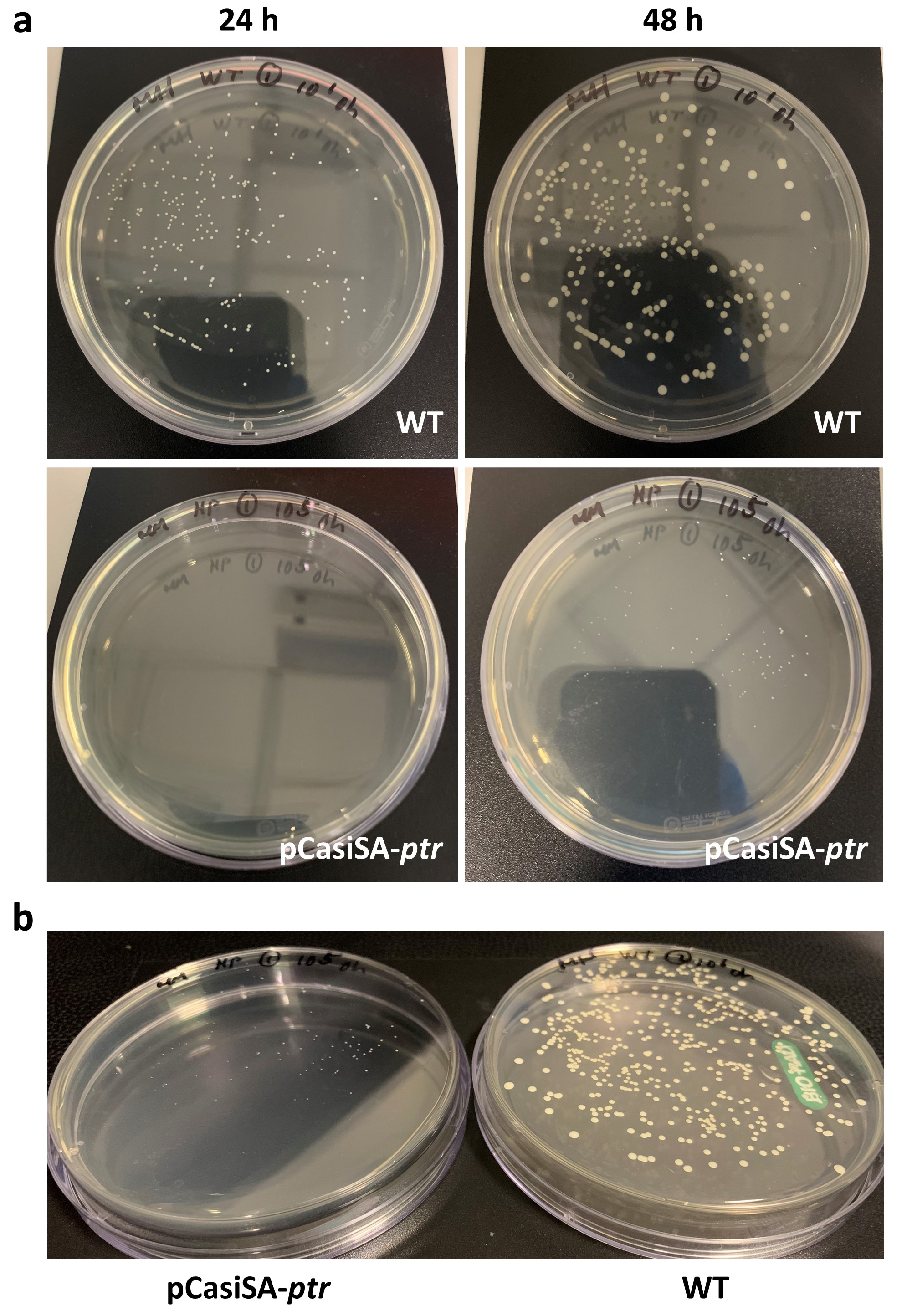

Supplement: FIG S3 [file msystems.01393-21-sf003.jpg]

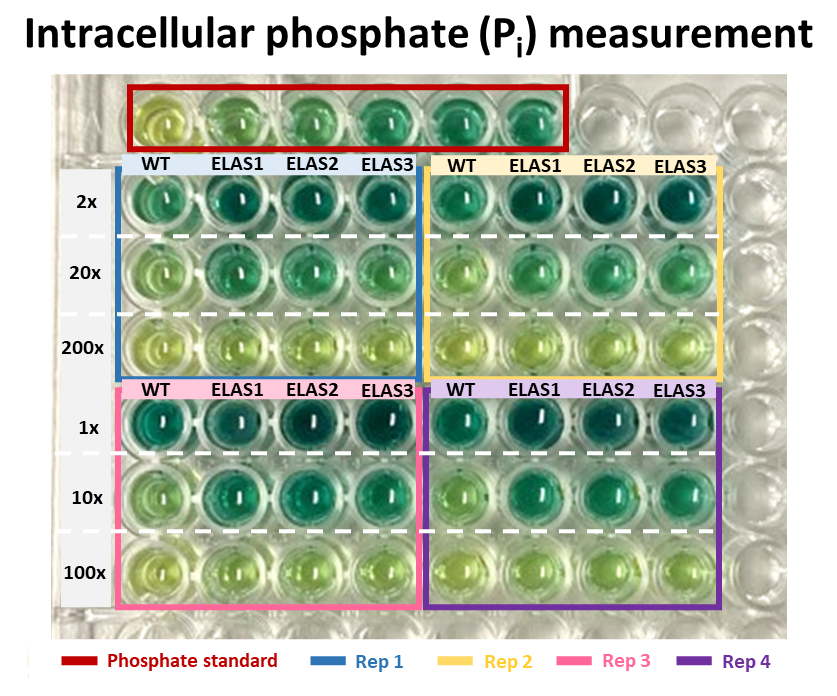

Supplement: FIG S4 [file msystems.01393-21-sf004.tif]

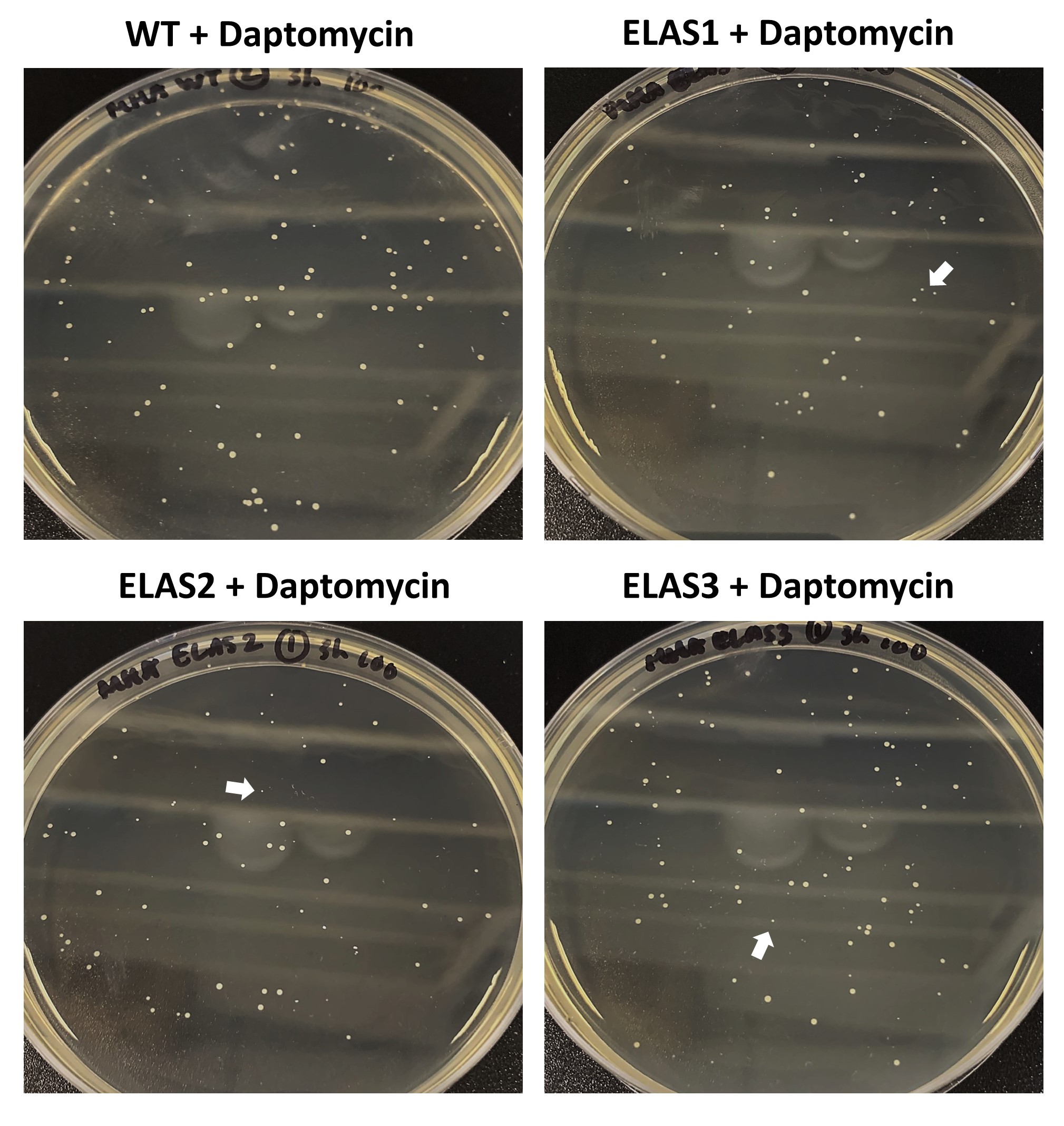

Supplement: FIG S5 [file msystems.01393-21-sf005.jpg]

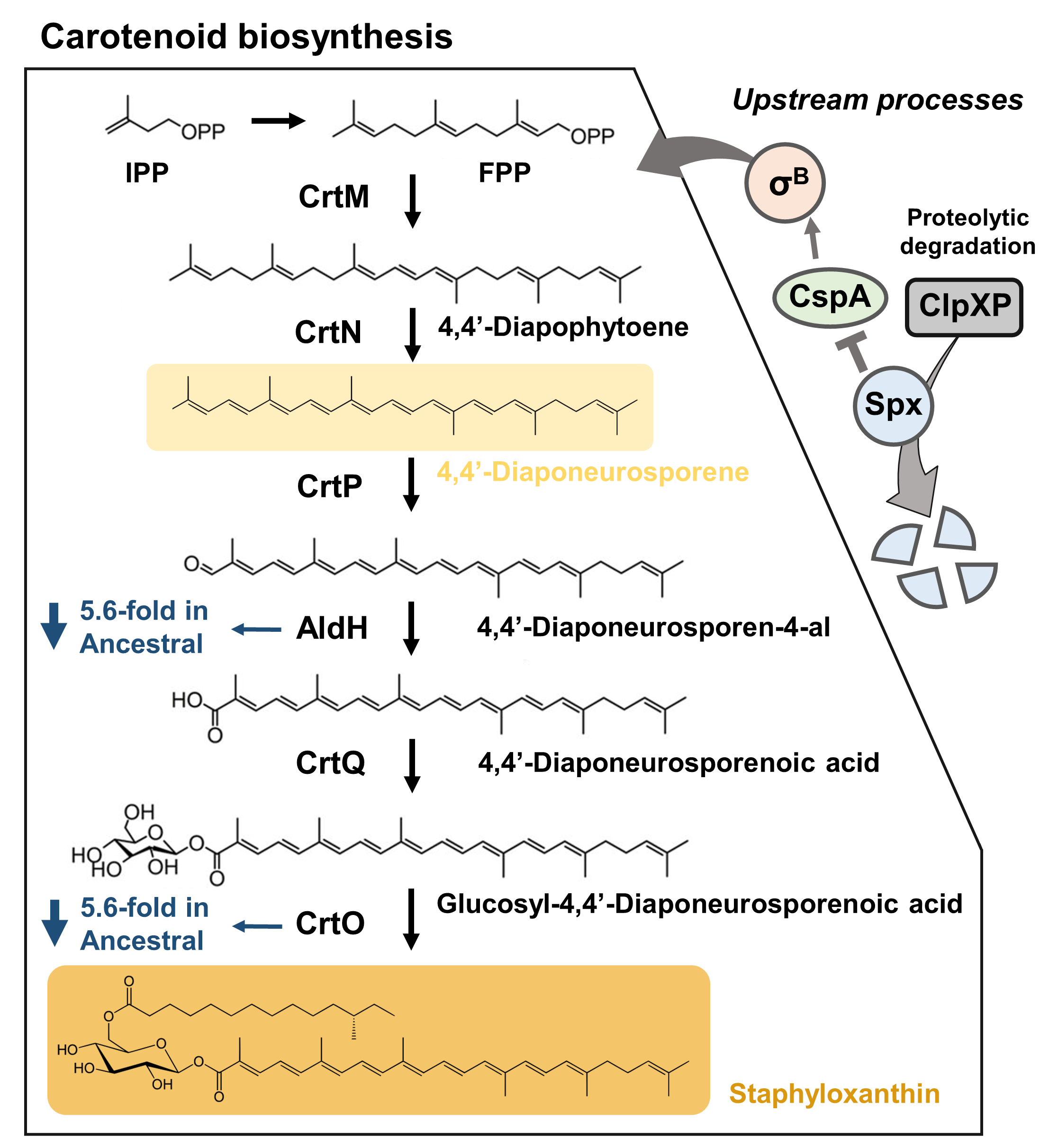

Supplement: FIG S6 [file msystems.01393-21-sf006.tif]
