# Supplementary material for: Proteomics and Transcriptomics Uncover Key Processes for Elasnin Tolerance in Methicillin-Resistant Staphylococcus aureus
Source: mSystems. 2022 Jan 25;7(1):e01393-21. doi: 10.1128/msystems.01393-21 (PMC8788329; doi:10.1128/msystems.01393-21)
Supplement: TABLE S1 [file msystems.01393-21-st001.docx]

| **Gene** | **Position/**  **mutation** | **Amino acid change** | **Protein annotation** | **Presence of mutation** | | |
| --- | --- | --- | --- | --- | --- | --- |
|  |  |  |  | **ELAS1** | **ELAS2** | **ELAS3** |
| *yjjP* | 1,290,910 /  A > C | N215H | Inner membrane protein YjjP | + | - | + |
| *hp* | 1,376,640 /  C > T | E149K | Hypothetical protein  (BLAST: DUF47 domain-containing protein/putative phosphate transport regulator) | + | + | + |
| *hisH* | 2,161,038 /  G > T | R182L | Imidazole glycerol phosphate synthase subunit HisH | - | + | - |
