# Supplementary material for: Proteomics and Transcriptomics Uncover Key Processes for Elasnin Tolerance in Methicillin-Resistant Staphylococcus aureus
Source: mSystems. 2022 Jan 25;7(1):e01393-21. doi: 10.1128/msystems.01393-21 (PMC8788329; doi:10.1128/msystems.01393-21)
Supplement: TABLE S4 [file msystems.01393-21-st004.docx]

| **Strains** | **Description** | **Reference** |
| --- | --- | --- |
| ***E. coli*** |  |  |
| DH5α | Host strain for plasmid | Invitrogen |
| **MRSA** | | |
| ATCC43300 | Wild-type methicillin-resistant *S. aureus* | ATCC |
| ATCC43300 pCasiSA | ATCC43300 with empty pCasiSA | This study |
| ATCC43300 pCasiSA-*ptr* | ATCC43300 with pCasiSA-*ptr* (phosphate transport regulator) | This study |
| **Plasmids** | **Description** | **Reference** |
| pCasiSA | transcription inhibition vector, Km^r^, Cm^r^ | Chen et al. (2017) *JACS*, 139(10):3790-3795 |
| pCasiSA-*ptr* | pCasiSA with *ptr* spacer | This study |
| **Primers** | **Sequence** | **Description** |
| pCasiSA-checkF | agaaaggcggacaggtatcc | For checking/ confirmation of pCasiSA |
| pCasiSA-checkR | ccgatagctaagcctattgag |  |
| *ptr-*spacer-F1 | GAAAgatcgtgcagctattgaatt | *ptr* spacer for pCasiSA |
| *ptr-*spacer-R1 | AAACaattcaatagctgcacgatc |  |
